# Safety and Efficacy of At-Home Robotic Locomotion Therapy in Individuals with Chronic Incomplete Spinal Cord Injury: A Prospective, Pre-Post Intervention, Proof-of-Concept Study

**DOI:** 10.1371/journal.pone.0119167

**Published:** 2015-03-24

**Authors:** Rüdiger Rupp, Daniel Schließmann, Harry Plewa, Christian Schuld, Hans Jürgen Gerner, Norbert Weidner, Eberhard P. Hofer, Markus Knestel

**Affiliations:** 1 Heidelberg University Hospital, Spinal Cord Injury Center, Heidelberg, Germany; 2 Ulm University, Institute of Measurement, Control and Microtechnology, Ulm, Germany; University of Regensburg, GERMANY

## Abstract

**Background:**

The compact **M**otorized orthosis for h**o**me **re**habilitation of **Gait** (MoreGait) was developed for continuation of locomotion training at home. MoreGait generates afferent stimuli of walking with the user in a semi-supine position and provides feedback about deviations from the reference walking pattern.

**Objective:**

Prospective, pre-post intervention, proof-of-concept study to test the feasibility of an unsupervised home-based application of five MoreGait prototypes in subjects with incomplete spinal cord injury (iSCI).

**Methods:**

Twenty-five (5 tetraplegia, 20 paraplegia) participants with chronic (mean time since injury: 5.8 ± 5.4 (standard deviation, SD) years) sensorimotor iSCI (7 ASIA Impairment Scale (AIS) C, 18 AIS D; Walking Index for Spinal Cord Injury (WISCI II): Interquartile range 9 to 16) completed the training (45 minutes / day, at least 4 days / week, 8 weeks). Baseline status was documented 4 and 2 weeks before and at training onset. Training effects were assessed after 4 and 8 weeks of therapy.

**Results:**

After therapy, 9 of 25 study participants improved with respect to the dependency on walking aids assessed by the WISCI II. For all individuals, the short-distance walking velocity measured by the 10-Meter Walk Test showed significant improvements compared to baseline (100%) for both self-selected (Mean 139.4% ± 35.5% (SD)) and maximum (Mean 143.1% ± 40.6% (SD)) speed conditions as well as the endurance estimated with the six-minute walk test (Mean 166.6% ± 72.1% (SD)). One device-related adverse event (pressure sore on the big toe) occurred in over 800 training sessions.

**Conclusions:**

Home-based robotic locomotion training with MoreGait is feasible and safe. The magnitude of functional improvements achieved by MoreGait in individuals with iSCI is well within the range of complex locomotion robots used in hospitals. Thus, unsupervised MoreGait training potentially represents an option to prolong effective training aiming at recovery of locomotor function beyond in-patient rehabilitation.

**Trial Registration:**

German Clinical Trials Register (DKRS) DRKS00005587

## Introduction

Loss of mobility has devastating effects on the quality of life of those affected and their ability to remain independent in the community. This applies to individuals with lesions of the central nervous system (CNS) sustained for example through stroke or spinal cord injury (SCI). In subjects with incomplete SCI (iSCI), intensive gait training leads to substantial improvements in walking function [1, 2]. The fundamental concept of motor function restoration is based on the notion that repeated execution of motor tasks induces plasticity—functional and structural reorganization of neuronal circuits—in the injured brain and spinal cord [3, 4]. A neuroanatomical structure that is particularly relevant for locomotion is the central pattern generator (CPG), which consists of a cluster of neuronal networks in the spinal cord [5–8] and is involved in the generation of stepping-like movements in supine and upright position in humans [9, 10]. In completely spinalized animals, the CPG can be trained by means of appropriate paradigms [11]. A series of recently conducted animal experiments introducing a chronological spinal cord dual-lesion paradigm revealed that in incomplete spinal lesions, adaptations of the CPG contribute to a larger extent to the recovery and re-expression of the locomotor pattern than was previously assumed [12]. To achieve a relevant level of independent locomotion, the CPG needs sufficient supraspinal input, [13] as well as afferent feedback from the peripheral nervous system [14].

Several factors of motor learning—task specificity, repetition, active participation and appropriate intrinsic and extrinsic feedback—have been identified as contributing to the long-term retention of a newly acquired skill [15, 16]. One clinical concept that successfully capitalizes on these principles is body weight-supported treadmill training (BWSTT) [17–19], which has been further transformed from manually assisted to automated BWSTT, including motor-driven gait orthosis [20] or specialized locomotion training devices [21]. While feasibility of robot-based locomotor training has been shown, its superiority to conventional gait training still has to be demonstrated [22]. Automated treadmill training substantially reduces therapists’ physical workload while also allowing the number of task-specific repetitions to be increased [23]. High-frequency task-oriented gait training requires substantial technical and personal support, which can primarily be provided by in-patient rehabilitation facilities. However, increasing economic constraints in health care require the duration of SCI in-patient rehabilitative treatment to be shortened [24]. Consequently, with earlier transition into out-patient and home-based therapy, the quantity and quality of rehabilitation training is dramatically reduced. Moreover, comparable studies in individuals with chronic iSCI suggest that long-term, mid-intensity locomotion training over a period of several months is more effective than application of high-intensity training protocols for only a few weeks [25, 26]. These facts underline the need to continue intensive locomotor training beyond in-patient rehabilitation.

Patients can most easily incorporate practice in their daily lives in a home-based training regimen. This may offer the advantage of practice within their personal space, where problem-solving is highly motivated [22]. Locomotion robotic systems may effectively support home-based training approaches by ensuring the generation of physiological movements and by providing objective feedback on training results. While the need for technically advanced locomotion therapy systems for home use is obvious, to date no such systems are available. A simple transfer of the existing devices to the patients’ homes is not possible, since most of them are restricted to application in a clinical or out-patient setting due to their size, weight and price. In addition, all of the devices have to be operated by skilled therapists. The main technical challenges of a home-based locomotion therapy device involve safety issues and its operation by the users themselves.

The **M**otorized orthosis for h**o**me **re**habilitation of **Gait** (MoreGait) is a robotic locomotion training device which has been developed and specifically designed for unsupervised, home-based therapy [27]. From a neurobiological point of view, it aims to promote neuroplasticity at different levels within the CNS: 1) It aims to generate important sensory stimuli, which were found to activate the CPG at the spinal level [14, 28, 29] and 2) it provides external feedback about the patient’s movement performance aiming at the compensation of the loss of sensation and/or proprioception and the enhancement of motor relearning at a supraspinal level [30]. From 2006 to 2008, 5 prototypes were built exclusively for research purposes and tested in several end users with iSCI [31].

The aim of this prospective, pre-post intervention proof-of-concept study was to test the safety of autonomous locomotor training with the MoreGait prototypes at the homes of individuals with sensorimotor iSCI and to obtain preliminary data about its efficacy. The pilot study results indicate that home-based robotic locomotion training with MoreGait is feasible and safe. The magnitude of functional improvements achieved by MoreGait training in individuals with iSCI is well within the range of complex locomotion robots used in hospitals.

## Study Participants and Methods

### Participants

For this prospective, baseline-controlled, single center cohort proof-of-concept study, inclusion criteria were (1) age between 18 and 60, (2) chronic (at least 1 year after trauma), (3) traumatic or ischemic/haemorrhagic sensorimotor iSCI (ASIA Impairment Scale (AIS) C, D [32]) and (4) with at least limited household ambulation (Walking Index for Spinal Cord Injury II [33] (WISCI II) > = 5). Exclusion criteria were body weight over 130 kg, height over 200 cm, contractures restricting the range of motion (ROM) to less than 80% of normal ROM, extreme spasticity, pressure sores, severe osteoporosis as well as any disease condition other than iSCI interfering with walking ability. Dropout criteria were the participant’s request to withdraw from the study and a weekly therapy intensity less than 4x 30 minutes. Study participants were identified by screening of the institutional database or were informed by advertisement on the institutional and other webpages. Additionally, a call for study participation was published in a magazine for people with disabilities focusing on individuals with SCI [34].

**Fig 1:**
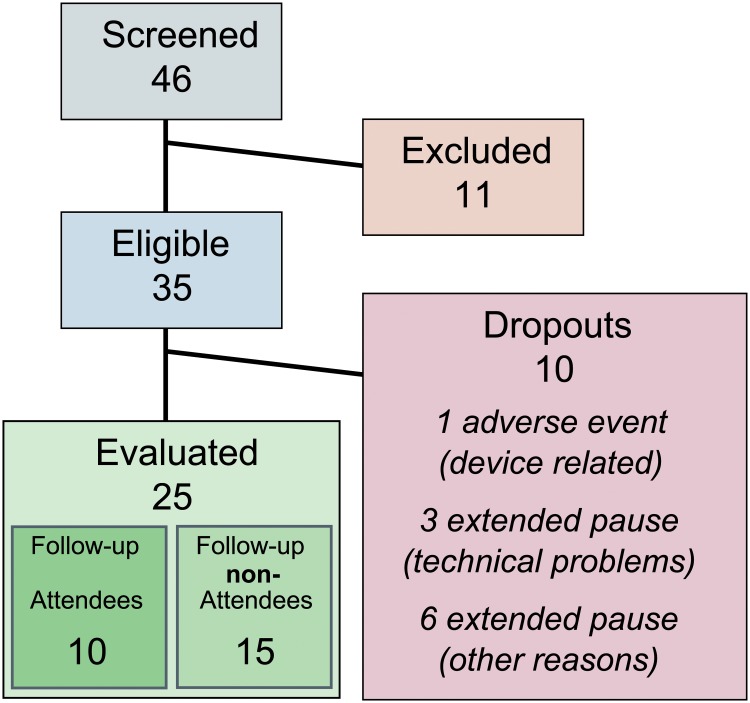
CONSORT flow diagram. The numbers of subjects involved in the different phases of the study are shown in the diagram.

Between January 2009 and January 2011, 46 individuals were screened, from whom 35 were included in the study. The intended number of study participants finalizing the training was set to 30 prior to the start of study due to the limited number of available prototypes and due to funding and time constraints. Twenty-five individuals (11 female, 14 male; 5 tetraplegic, 20 paraplegic; mean age: 44.0 ± 12.4 (SD) years) with chronic (mean time since injury: 5.8 ± 5.4 (SD) years) sensorimotor iSCI (7 AIS C, 18 AIS D; WISCI II from 5 to 19) completed the training procedure (Fig. 1). Ten of these twenty-five individuals who completed the training took part in the follow-up assessment.

### Ethics statement

The study was approved by the Ethics Committee of Heidelberg University Hospital (vote no. MV-174/2007) and was conducted according to the World Medical Association Declaration of Helsinki and the Guidelines for Good Clinical Practice. The protocol submitted to the ethical committee for this clinical trial and supporting TREND checklist are available as supporting information (see S1 TREND Checklist and S1 Protocol).

**Fig 2:**
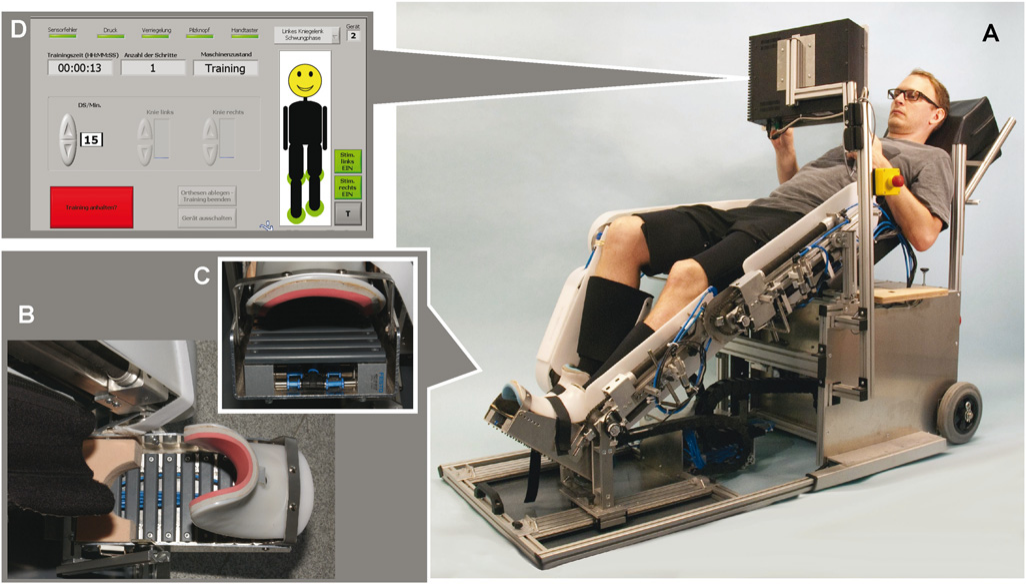
Overview of the components of the MoreGait device. A subject during training in the MoreGait device (A), top (B) and front (C) view of the mediolateral bars of the stimulative shoe, user interface and feedback screen (D).

It has been registered at the German Institute for Medical Documentation and Information (DIMDI) as a clinical trial (registration no. 9053) with a novel medical product according to the guidelines of the European Medicinal Devices Act and with the main ID DRKS00005587 in the the German Clinical Trials Register (DKRS). Participants gave written informed consent prior to study inclusion. The individual shown in Fig. 2 of this manuscript has given written informed consent (as outlined in PLOS consent form) to publish his case details.

### Robotic device

The MoreGait device used in this study consists of a special seat combined with an inclined backrest, a pneumatically driven gait orthosis for each side to assist movements of both legs in the sagittal plane (independently driven knee and ankle joint, hip joint mechanically linked to knee joint via a fixed kinematic chain that allows only horizontal movements of the ankle joint) and a dedicated mechanical foot stimulation unit. Its dimensions are 172 x 70 x 130 cm (l x w x h) and the total weight is approximately 115 kg (Fig. 2A).

Pneumatic fluidic muscles (Festo AG & Co. KG, Esslingen, Germany) were selected as actuators. They offer the advantage of inherent low stiffness, which results in soft, safe and comfortable movements. Additionally, control parameters were set to allow for deviations of up to 5° from the predefined movement trajectory [35, 36], which permits the user to explore different muscular activation strategies to follow the reference trajectories.

For safety reasons, the user’s body is placed in a semi-supine position. In this configuration, sufficient loading of the foot sole during stance phase cannot be generated by the user’s own body weight. Therefore a novel device—a “stimulative shoe”—was developed to mimic the loading of the foot sole without requiring the patient to be completely verticalized. This mechanical stimulation unit consists of 10 mediolateral plastic bars, which are mounted on pairs of pneumatically driven short-stroke cylinders (Fig. 2B and Fig. 2C). The generated foot loading reaches approx. 30% body weight. The timing of actuation with a sequential stance-phase related cylinder activation sequence starting at the heel and ending in the toe region and the force of each pair of cylinders can be set by software, which forms the basis for generating a physiological loading pattern. The stimulative shoe is by design also capable of generating artificial loading patterns such as gait-phase related vibrational stimuli. However, in its current version the mobile compressor does not provide enough air-mass per minute to apply sufficient force in vibration mode. For this reason, the initial plan to randomly assign the study participants into two groups, who receive a therapy either based on physiological or vibrational loading of the foot sole, was abandoned.

One of the key factors for any kind of locomotion therapy to succeed is active participation by the patients. In order to continuously provide the users with information about whether they are performing training correctly, a feedback functionality was implemented. A rating measure is calculated from the user’s estimated active torques, and both the progress of the training and the absolute performance level are visualized on a display (Fig. 2D).

The right orthosis as well as the backrest can be lowered manually, enabling the patient to transfer autonomously to the lowered backrest. After successful transfer, the backrest can be inclined and the orthosis can be lifted in the training position. Leaving the device is performed in reverse order.

### Study protocol

**Fig 3:**
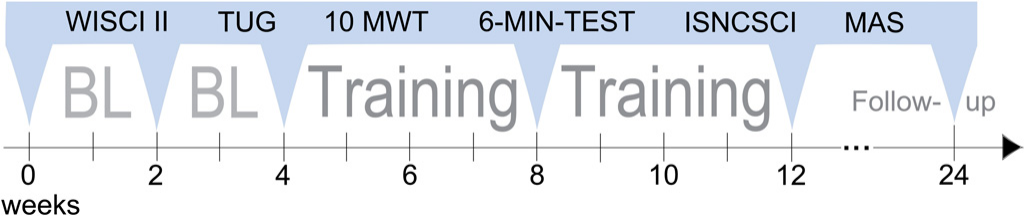
Study protocol showing the course of the study and its assessments on a timeline. Three baseline (BL) assessments within the first 4 weeks are followed by 2 assessments at the middle and end of the training period. The follow-up assessment was carried out 3 months after the end of training. Assessments included the Walking Index for Spinal Cord Injury II (WISCI II), Timed Up and Go Test (TUG), 10-MeterWalk Test (10MWT) at both self-selected and maximum speed, six-minute walk test (6-MIN-TEST), as well as International Standards for Neurological Classification of Spinal Cord Injury (ISNCSCI) assessments and measurement of spasticity according to the modified Ashworth scale (MAS).

After screening and study inclusion, 3 assessment visits(0, 2 and 4 weeks) within a 4-week baseline period were planned prior to training onset, followed by assessments in the middle and at the end of an 8-week training period and a follow-up assessment 3 months after the end of training (Fig. 3) [37]. Assessments were performed by unblinded examiners either at the Spinal Cord Injury Center of Heidelberg University Hospital or at the study participants’ homes. Baseline assessments and follow-up assessments were done at the Spinal Cord Injury Center, whereas the majority of 4-week and 8-week assessments were conducted at the participants’ homes.

The assessments of each visit consisted of a set of well-established functional and neurological tests. The assessment scheme and outcome parameter were defined prospectively before the start of the study and was not changed over the course of the study. The self-selected WISCI II was defined as the primary outcome measure of the study due to its importance for the participants [38]. The WISCI II is a 21-item ordinal scale that classifies dependency on walking aids over a distance of 10 meters (0 = not able to walk 10meters, 20 = walking without help for 10 meters). For quantification of walking ability, the 10-Meter Walk Test (10MWT) at self-selected speed (10MWT(sss)) as well as maximum speed (10MWT(ms)), the Timed Up and Go Test (TUG) and the six-minute walk test (6-MIN-TEST) were applied [39, 40]. The 10MWT measures the time required to walk 10 meters and quantifies the short-duration walking speed. The TUG measures the time required for a patient to stand up from an armchair, walk 3 meters, return to the chair, and sit down. The 6-MIN-TEST measures the distance walked within 6 minutes and serves as a measure for endurance. All walking tests were performed with the participants using their currently appropriate walking aid(s) on even and straight walkways. If tests were performed at the participant’s home, walking tests were done on barrier free, even grounds, like sports fields or non-crowded roads.

Dermatomes and myotomes as defined by the International Standards for Neurological Classification of Spinal Cord Injury (ISNCSCI) [41] were examined to check for spontaneous neurological recovery during baseline. ISNCSCI’s lower extremity motor score (LEMS) was used to specifically assess the changes in strengths of the key muscles of the legs during the training period. A high-quality ISNCSCI assessment was assured by trained assessors [42] and computer-based scaling, scoring and classification of the ISNCSCI [43]. To determine the degree of spasticity in hip, knee and ankle joints in terms of increased resistance against passive movements in the sagittal plane, the modified Ashworth scale (MAS) was administered [44].

### Training

During the 8-week therapy period, individuals trained with the MoreGait device for 30–45 minutes per day, 4 to 6 days per week. Users were instructed to set step frequency at a comfortable level to avoid fatigue during each session. The training took place at the participants’ homes without supervision of the study personnel (mean distance from the spinal cord injury center in Heidelberg 209.12 ± 162.16 km; Google Maps (http://maps.google.com), fastest route). Prior to the first training session, each participant was familiarized with proper usage of the MoreGait machine. The 30-minute instruction included practicing the transfer procedure for getting into and out of the device and securing the thigh and shank straps to the legs, along with explaining the elements of the user interface displayed on the touch screen together with the correct selection of therapy parameters. During a short trial session, an experienced technician or scientist adjusted the length of the machine’s linkages and position of the orthotic fittings in order to ensure correct alignment of technical and anatomical joints. To help isolate the effect of the training device, participants were instructed not to modify their regular physical therapy, unsupervised training program, or antispastic medication during the study period.

### User survey

To assess the users’ satisfaction with technical design, safety and therapeutic functionality of the MoreGait device, a user survey was made. The survey consisted of a paper questionnaire which was sent to each study participant who finished the 8-weeks of therapy. The proprietary questionnaire consisting of 51 questions was designed for obtaining dedicated user feedback on details of the MoreGait device. It mainly used a five-point scale for rating of answers, which is known from other standardized surveys on assistive technology [45], supplemented by Yes/No questions and comment fields.

Twenty of the twenty-five study participants, who completed the 8-weeks MoreGait training, replied to the survey. The results were grouped into the three main categories “Therapy outcome”, “Transfers, fastening and release” and “Training experience” including perception of safety. Results were analyzed on a descriptive basis eg, with boxplots.

### Statistical analyses

Statistical analysis was performed with R 2.15.1 [46]. Friedman’s test was used to test for overall significance of differences between all assessments, including baseline (BL1, BL2, BL3) assessments. For statistical analysis, raw values were used. Significance level was set to α = 0.05. Pairwise Wilcoxon-Mann-Whitney tests were used for post hoc comparisons between all assessment visits, including baseline visits. To address the multiple testing problem, Benjamini-Hochberg-corrections [47] were applied. The 4-week and 8-week assessments were compared separately with each of the three baseline assessments. Differences to baseline were considered significant, if the mean of the 3 p-values from the comparison of each baseline assessment with the respective therapy assessment was $\bar{P}$ < 0.05. An overview of all p-values and confidence intervals is provided in S1 Table. As non-parametric tests were used, confidence intervals (ci) are intervals of the differences of the location parameters.

Baseline stability was tested in the context of the Friedman’s test. The baseline is considered stable, if pairwise post hoc tests of all three baseline assessments are not significant.

If not stated otherwise, numbers are displayed as mean ± SD. To allow for a more generalized analysis of the assessments of different study participants at 4 and 8 weeks of therapy, percentage values are calculated which are normalized to the mean baseline representing 100%.

The follow-up analysis was reduced to descriptive statistics because of the low number of available datasets for this stage (N = 10). Data from individuals that did not complete the study were not included in the analysis.

## Results

### Outcomes on safety and dropouts

One device-related adverse event (Grade 2–3 [48] pressure sore on the tip of the left big toe) caused 1 participant to drop out. The pressure sore was caused by a pressurized bar of the stimulative shoe, which applied its full load to the tip of the big toe. It healed after discontinuation of therapy and wound dressing for 2 weeks. After implementation of a foot length-dependent deactivation of most anterior mediolateral bars of the stimulative shoe, no further adverse events occurred. Other reasons for dropouts were prolonged periods of non-use, such as those resulting from bladder infections or technical problems in combination with delayed repair due to great distances between the users’ homes and the authors’ institutions (Fig. 1). Individuals who did not attend the follow-up assessment, but completed the training were not considered as dropouts.

### Stability of baseline

Baseline stability was shown for all outcome measures, since all baseline post hoc comparisons were not significant (see S1 Table for detailed information). During baseline, three temporary conversions occurred (AIS C to B and back to C, AIS C to D and back to C, and AIS D to C and back to D).

### Outcome measures during therapy

**Fig 4:**
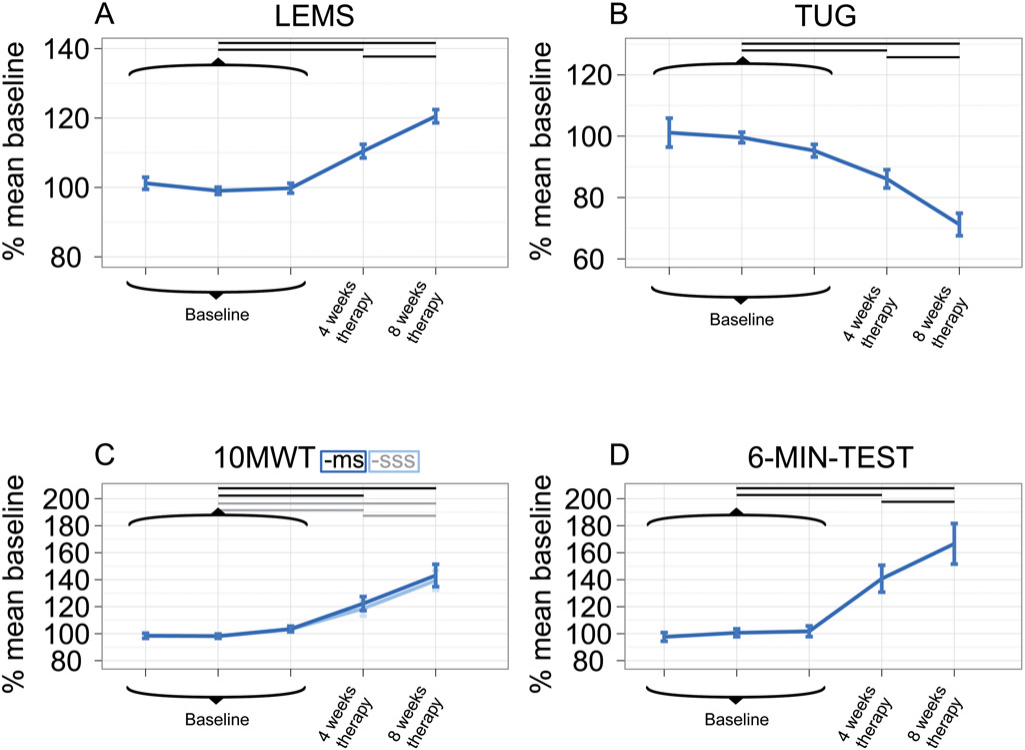
Overview of all study visits in relation to mean baseline. Percent of the mean baseline ± standard error (SE) along the course of the study in relation to the mean baseline for (A) Lower extremity motor score (LEMS), (B) Timed Up and Go Test (TUG), (C) 10-MeterWalk Test (10MWT) at maximum speed (-ms) and self-selected speed (-sss), (D) six-minute walk test (6-MIN-TEST). Horizontal bars mark significant differences (p < 0.05), which were determined on the basis of the absolute values in Table 1.

A numerical overview of the absolute outcome measures is given in Table 1, while Fig. 4 presents the course of the changes in the walking test results relative to baseline as a graph.

**Table 1:** Numerical summary of outcome measures from 6 visits.

Visit	Baseline 1	Baseline 2	Baseline 3	Mean / Median Baseline*	4 weeks therapy	8 weeks therapy	8 weeks therapy^b^, *N = 10*	Follow-up(20 weeks)^b^, *N = 10*
Outcome measure								
WISCI II [score]^a^	12 (9–16)	12 (9–16)	12 (9–16)	12 (9–16)	16 (11.75–16); {24}	16 (15–16);{24}	16 (12–18.25)	14 (11.25–16)
MAS [score]^a^	2 (0–9)	1 (0–4)	2 (0–4.5); {24}	1 (0–6.33)	0 (0–6)	1 (0–4.5); {24}	0 (0–8.25)	1 (0–7.0)
10MWT (sss) [m/s]	0.37 ± 0.27	0.36 ± 0.24	0.37 ± 0.24	0.37 ± 0.25	0.42 ± 0.30;{24}	0.47 ± 0.27;{24}	0.52 ± 0.35	0.59 ± 0.42
10MWT (ms) [m/s]	0.47 ± 0.43	0.45 ± 0.35	0.46 ± 0.37	0.46 ± 0.38	0.52 ± 0.42; {24}	0.57 ± 0.35; {24}	0.66 ± 0.46	0.71 ± 0.49
6-MIN-TEST [m]	117.04 ± 103.87; {24}	115.63 ± 103.08; {24}	119.46 ± 109.78; {24}	117.38 ± 104.59	142.74 ± 107.01; {23}	164.74 ± 115.03; {23}	185.1 ± 154.88	210.70 ± 162.96
TUG [s]	67.71 ± 57.89; {24}	61.60 ± 51.62	56.18 ± 38.34	61.34 ± 48.02	50.83 ± 36.10; {24}	37.21 ± 22.71; {24}	37 ± 25.36	50.80 ± 54.96
LEMS [points]	30.32 ± 9.33	29.72 ± 8.99	29.92 ± 9.24	29.99 ± 8.97	33.36 ± 10.87	36.42 ± 9.77; {24}	35.1 ± 11.28	33.70 ± 11.49
MS L2 [points]	7.48 ± 1.05	7.32 ± 1.35	6.84 ± 1.46	7.21 ± 1.13	7.76 ± 0.93	7.88 ± 1.20	8.1 ± 1.37	8.20 ± 1.69
MS L3 [points]	7.60 ± 1.32	7.36 ± 1.70	7.48 ± 1.42	7.48 ± 1.24	8.40 ± 1.58	8.52 ± 1.56	9.1 ± 0.99	8.60 ± 1.51
MS L4 [points]	4.84 ± 3.21	4.68 ± 3.22	5.04 ± 3.36	4.85 ± 3.14	5.80 ± 3.56	6.48 ± 3.33	6.7 ± 3.30	6.00 ± 3.37
MS L5 [points]	5.04 ± 3.61	4.96 ± 3.48	5.00 ± 3.42	5.00 ± 3.44	5.44 ± 3.63	6.08 ± 3.98	5.1 ± 4.58	5.00 ± 4.55
MS S1 [points]	5.16 ± 3.22	5.32 ± 3.18	5.60 ± 3.11	5.36 ± 3.02	6.24 ± 3.46	6.80 ± 3.34	6.1 ± 3.87	5.90 ± 4.01

Mean ± standard deviation of Walking Index for Spinal Cord Injury II (WISCI II), modified Ashworth scale (MAS), 10-Meter Walk Test (10MWT)—self-selected speed (sss) and maximum speed (ms), six-minute walk test (6-MIN-TEST), Timed Up and Go Test (TUG) and lower extremity motor scores (LEMS) are listed chronologically. Segmental motor scores (MS) for myotomes L2—S1 are also provided. Sample sizes are displayed in “{}” where they deviate from N = 25.

^a^Median and 25%-75% quartiles are given for ordinal scales eg, WISCI II and MAS.

^b^As follow-up data have a sample size of N = 10 the corresponding subset of patients within the 8-week therapy assessment is presented for better comparison.

The primary outcome measure, WISCI II, showed overall significant results (p < 0.0006) and increased from 12 (9 to 16) to 16 (15 to 16) (baseline to 8 weeks therapy; median (interquartile range (IQR))). Nine participants (2/7 AIS C, 7/18 AIS D) were less dependent on walking aids after therapy than before. A qualitative analysis of the AIS subgroups revealed that subjects classified as AIS C show a trend towards a higher improvement than those classified as AIS D (median of increase from baseline to end of therapy in AIS C = 7 and in AIS D = 4). Post hoc tests in WISCI II revealed a significantly ($\bar{P}$ = 0.0352, ± 0.0035) higher WISCI II level after 8 weeks of training compared to baseline, with a positive tendency ($\bar{P}$ = 0.1470, ± 0.0504) already identified after 4 weeks.

The results of the 10MWT for both self-selected (139.4% ± 35.5%; 8-weeks assessment) and maximum (143.1% ± 40.6%; 8-weeks assessment) speed conditions showed a significant overall improvement (both p < 0.0001) in short-distance walking velocity. Post hoc analysis revealed a significant increase of self-selected ($\bar{P}$ = 0.0015 ± 0.0006) and maximum ($\bar{P}$ = 0.0042, ± 0.0054) walking speed between baseline and end of therapy. Moderate improvements are already visible between baseline and 4-weeks assessments ($\bar{P}$ = 0.0317 ± 0.0156 for self-selected speed and $\bar{P}$ = 0.0497 ± 0.0269 for maximum speed). Moreover, self-selected speed increased significantly (p = 0.0227) between week 4 and 8. Endurance estimated with the 6-MIN-TEST (overall significance p < 0.0001) showed significant improvements in post hoc tests at the end of therapy (166.6% ± 72.1%, $\bar{P}$ = 0.0006 ± 0.0004), as well as during the first half ($\bar{P}$ = 0.0019 ± 0.0006) and the second half of the therapy period (week 4 to week 8, p = 0.0026). Outcomes for the TUG were overall significant (p < 0.0001). Time needed to perform the TUG decreased considerably to 71.2% ± 18.0% over the course of the 8 weeks of therapy ($\bar{P}$ = 0.0004 ± 0). Significant differences in the time needed to perform the TUG were also found between baseline and 4-weeks assessment and 4-weeks and 8-weeks assessment ($\bar{P}$ = 0.0097 ± 0.0132 and p = 0.0004, respectively).

There were significant overall results in LEMS (p < 0.0001). Post hoc analysis showed significant ($\bar{P}$ = 0.0001 ± 0) increases in LEMS throughout the entire training phase, up to 120.5% ± 9.3% at the 8-weeks assessment. Post hoc analysis revealed a significant improvement in the strength of key muscles from baseline to 4 weeks ($\bar{P}$ = 0.0002 ± 0.0001) and 8 weeks ($\bar{P}$ = 0.0001 ± 0) of therapy and between 4 and 8 weeks of therapy (p = 0.0054).

The MAS of all study participants did not show any significant differences over the course of the therapy (p = 0.2379). However, in the 7 participants in whom spasticity was present (defined by a mean MAS > 4) at baseline (median at baseline: 16 (IQR 9.5 to 16)), a trend towards decreased spasticity (median: -3 (IQR-5.5 to -1.5)) was observed.

Two temporary conversions in the AIS (B to C and back to B and D to C and back to D) were detected during the therapy (onset, 4 weeks, 8 weeks) period [49]. The neurological level of injury remained within ± 2 segments referenced to baseline in all participants.

### Follow-up examinations

**Fig 5:**
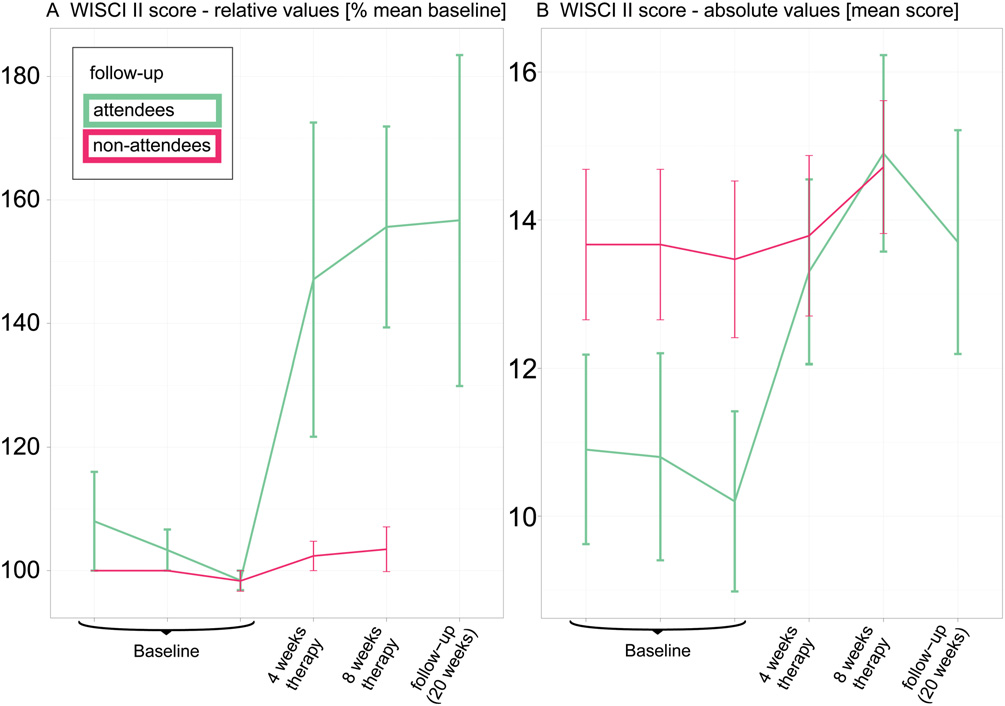
Analysis of the Walking Index for Spinal Cord Injury II (WISCI II) scores of the attendees of the follow-up visits. WISCI II scores displayed as (A) relative values ± standard error (SE) and (B) absolute values ± SE for participants (N = 10) who attended the follow-up assessment (green) and for participants (N = 15) who did not attend the follow-up assessment (red).

Baseline and follow-up assessments (Fig. 3) were scheduled at the spinal cord injury center, while tests performed after 4 and 8 weeks of training were conducted at the participants’ homes. Accordingly, the follow-up assessment was associated with significant travel efforts for the participants. Ten individuals attended the follow-up assessment. We performed a non-confirmatory descriptive subgroup analysis to search for differences in outcome measures between the groups of “follow-up attendees” and “non-attendees”. Differences between these subgroups were found only in the WISCI II assessment with attendees of the follow-up visit showing 1) a much higher relative improvement at end of therapy (Fig. 5A) and 2) a much lower absolute baseline level than non-attendees (Fig. 5B).

Analysis of the WISCI II follow-up assessments shows that after the end of therapy, 1 subject further improved (9 to 16), 7 remained at the same level and 2 became worse (19 to 5, 16 to 11). Five months after therapy onset, 7 of the 10 follow-up visitors were less dependent on walking aids compared to baseline.

### Patient reported outcome

**Fig 6:**
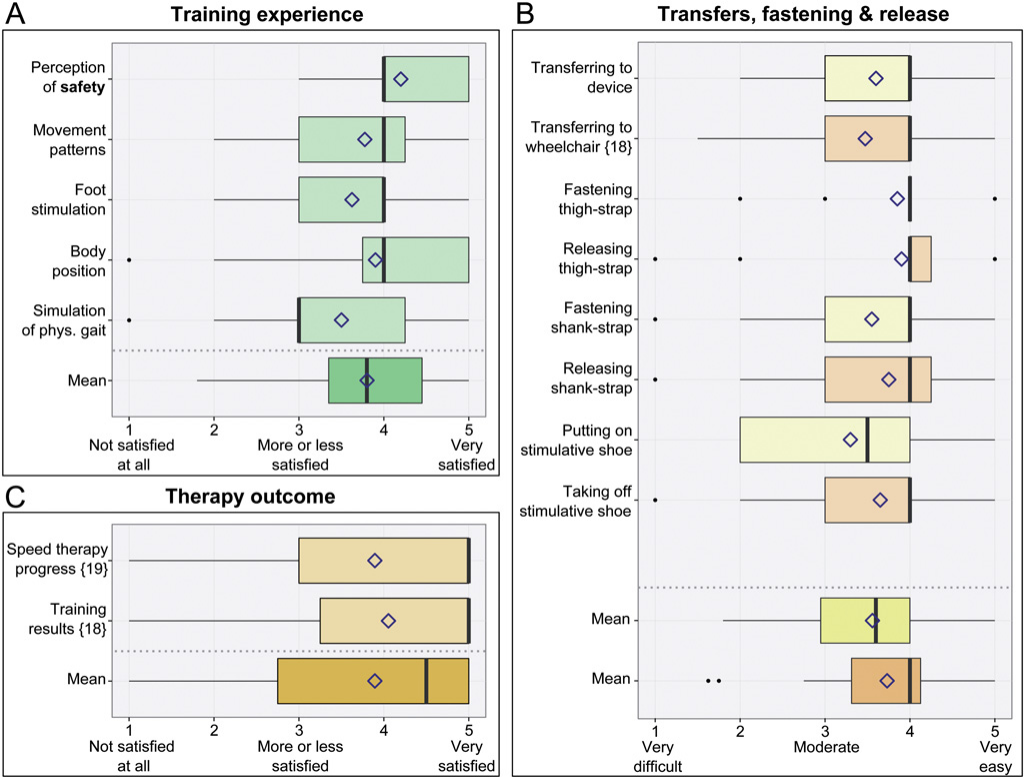
User survey results. Boxplots showing survey results on a 5 point scale for the categories (A) Training experience, (B) Transfers, fastening and release, and (C) Therapy outcome. The survey was completed by twenty of the twenty-five participants who finished the MoreGait study. Sample sizes are displayed in “{}”, where they deviate from N = 20.

Individuals were overall satisfied with their training experience (3.80 ± 0.85, Fig. 6A). The perception of safety during training was rated high, with a mean score of 4.20 ± 0.77. While movement patterns, foot stimulation and body position during training were rated as good, the simulation of physiological gait was rated as moderate.

Transfers on the device and back to the wheelchair, as well as fastening and releasing leg straps were rated as between moderate and easy (Fig. 6B), whereby transfer and fixation on the device were rated as to be slightly more difficult (3.58 ± 0.87) than releasing the straps and transfer back to the wheelchair (3.73 ± 1.0). Five individuals, among them two individuals with tetraplegia, declared the need for help in the process of mounting and/or dismounting the device.

Study participants were overall satisfied with the outcome of the therapy (Fig. 6C), indicated by a mean satisfaction score for the category “Therapy outcome” of 3.89 ± 1.49.

## Discussion

We investigated the safety and efficacy of 5 prototypes of the novel MoreGait robotic locomotor training device, which has been explicitly developed and designed for autonomous use in the home environment. This is to our knowledge the world’s first locomotor training device dedicated to this purpose [50].

We found that home-based training with this compact device is feasible and effective, and could be handled well by the users. A study assessing the safety of the supervised application of the driven gait orthosis Lokomat in children and adolescents reported 5 adverse events requiring discontinuation of therapy in about 1.400 training sessions [51]. In more than 800 unsupervised training sessions of comparable duration with the MoreGait only one device-related adverse event occurred. In conclusion, the low incidence of device-related adverse events, the non-occurrence of any serious adverse event and the overall positive patient reports on safety and usability show that the MoreGait can be used safely and mainly independently in the home environment by sensorimotor impaired end users without continuous supervision by clinical experts.

The participants in our study with chronic iSCI showed no signs of neurological or functional recovery during the 4-week baseline period. This clearly demonstrates that the documented improvements following MoreGait training were therapy related and not spontaneous. The mean gain of 2.08 ± 3.82 levels in WISCI II clearly exceeds the recently reported clinically meaningful threshold of 1 level in iSCI [52].

The users benefited from the home-based robotic locomotion therapy in a variety of ways. The dependency on walking aids—a highly relevant issue for individuals with iSCI—was remarkably reduced in 9 participants after the MoreGait therapy. The importance of this aspect for the study participants is underlined by the outcome of the descriptive subgroup analysis of follow-up attendees vs. non-attendees. Attendees of the follow-up assessment had a much higher increase in WISCI II levels than non-attendees. The therapy-induced gain in WISCI II levels and *not* the absolute level at the end of therapy appears to influence participants’ motivation level and, as a consequence, their willingness to participate in the last study assessment. The fact that the MoreGait therapy was home-based might have a negative impact on the general willingness to follow-up attendance compared to studies with inpatient interventions. However, to confirm the results from this descriptive subgroup analysis, a higher number of study participants needs to be included.

During the therapy period, the participants’ walking ability increased considerably. This was reflected by improvements in short-distance gait speed (10MWT) by approximately 40%, endurance (6-MIN-TEST) by roughly 65% and standing up, turning, and sitting down (TUG) by around 30%. Those improvements were also seen in the 9 individuals, who needed less support by walking aids over the course of the therapy.

A direct comparison of the study results with other studies is very difficult due to differences in patient populations (type/severity of lesion, functional status, exclusion of spontaneous recovery,) and therapy regimens (frequency, duration) [53, 54]. However, to allow for direct comparison of results, the inclusion criteria and protocol selected for the MoreGait study were similar to those used for a study with the Lokomat [26]. The extent of improvement in walking speed and endurance achieved by MoreGait is comparable to that of supervised clinical gait rehabilitation with the Lokomat (10MWT: 56%, 6-MIN-TEST: 53%, TUG: 32%). However, a higher percentage of study participants was less dependent on walking aids after MoreGait training (MoreGait: 9/25, Lokomat: 2/21). Interestingly, participants in the MoreGait study increased gait speed and endurance during the therapy period in nearly linear fashion, indicating that MoreGait training performed for longer than 8 weeks might further improve locomotor function. Harkema et al. assessed the effects of intensive locomotor training, including step training using body-weight support and manual facilitation on a treadmill followed by overground assessment and community integration, in 196 individuals with chronic iSCI (approximately the same percentage of one third AIS C and two thirds AIS D participants as in our study) who underwent at least 20 locomotor training treatment sessions in outpatient rehabilitation centers [55]. Although the number of therapy sessions varied extensively, the group of chronic patients assessed within 1 to 3 years after trauma improved on average 0.11 ± 0.23 m/s in the 10MWT and 44 ± 71 m in the 6-MIN-TEST. Our participants improved to a similar extent (10MWT: 0.1 m/s; 6-MIN-TEST: 47 m), although they were not explicitly supervised by experienced therapists.

A number of studies utilizing body weight-supported training for improving walking in individuals with SCI have reported improvements in lower-limb strength in patients with chronic SCI that are in the range of our results [56, 57]. The improvements in walking ability could potentially be attributed only to the significant increase in LEMS. However, the motor scores of the proximal muscle groups, which are highly relevant for walking function, did not increase noticeably in the second half of the training period. Yet, locomotor function continued to improve to a similar extent compared to the first 4 weeks of training (Table 1). This points to an improvement in coordination rather than in muscle strength. Interestingly, the improvements in distal muscle strength, which mainly contributed to the increase in the total LEMS, are in the same range within the first and second half of the therapy period, indicating the high therapeutic relevance of the stimulative shoe.

There was a trend towards decreased spasticity at the end of therapy in study participants with a mean MAS at baseline greater than 4. However, this finding has to be interpreted very carefully due to the low reliability of the MAS to detect subtle changes in spasticity [58].

The following limitations of the study have to be considered: Besides screening of the internal medical database, study participants were recruited by advertising the study on the university hospital’s website and in a magazine for people with disabilities focusing on individuals with SCI [34]. The recruitment procedure, together with the lack of reimbursement of travel expenses, may have contributed to a selection bias towards exceptionally motivated individuals. Considering the high dropout rate, the criterion for dropping out—less than 4x 30 minutes therapy time per week—was most likely too ambitious.

We did not document the type and focus of concomitant therapies and medication. Although study participants were asked not to modify their physical therapy, unsupervised training program, or antispastic medication, it cannot be excluded that changes in the regimes of these therapies throughout the MoreGait training period contributed to the improvements. The MoreGait therapy was applied at home as an add-on therapy. Therefore, we cannot exclude that the gait improvements were simply caused by the higher training intensity. On the other hand, this regimen of use best reflects the intended application scenario, in which MoreGait is the key component for allowing a higher intensity of gait training at home. Randomized controlled studies are necessary in the future to show the superiority of the MoreGait training in comparison to other, more simple home-based therapies.

Due to the safety-driven design of the MoreGait the user is put in a semi-reclined position. The influence of this non-physiological posture during the locomotion therapy with MoreGait on balance needs to be determined in future studies.

While baseline and follow-up assessments were performed at the Spinal Cord Injury Center, the majority of the 4-weeks and 8-weeks assessments took part in the participant’s home environment. While no influence of a community environment is reported on the 10MWT, positive effects are described on the gait endurance assessed by the 6-MIN-TEST [59]. This may lead to a systematic bias in the results of the 6-MIN-TEST obtained during the therapy period.

The findings of the present study demonstrate that a robotic device reduced to a technical minimum can be introduced into a feasible, safe and effective gait rehabilitation therapy at home and thus might influence future robotic gait-rehabilitation strategies. A randomized-controlled trial investigating the effects of MoreGait therapy in acute iSCI is currently underway. Other neurological disease conditions affecting locomotor function may also benefit from this kind of robotic therapy, and thus warrant future investigation. The MoreGait device represent a valuable platform for future investigations on systematic identification and ranking of the therapeutic impact of machine parameters like degree of foot loading, inclination of the backrest or the prolonged therapy time.

## Conclusions

Robotic home-based locomotion therapy with MoreGait allows patients to continue high-frequency training of locomotor function based on principles of activation of spinal locomotor networks and of motor learning after discharge from rehabilitation centers. The functional improvements following 8 weeks of MoreGait therapy in individuals with chronic sensorimotor iSCI are well within the range of those achieved with complex locomotion robots used at hospitals [26]. The stimulative shoe provides the opportunity to investigate alternative foot-loading patterns (eg, gait phase-related vibrational patterns), which might be even more effective in activating the spinal locomotion network [28]. Of course, other neurological diseases affecting locomotor function may also benefit from this kind of robotic therapy, and thus warrant future investigation.

## Supporting Information

S1 TREND Checklist TREND statement checklist. (PDF)

S1 Protocol Trial Protocol. (PDF)

S1 Table P-values and confidence intervals. Overall p-values, as well as p-values and confidence intervals (ci) of the post-hoc comparisons of all baseline (BL) measurements, each BL with 4 weeks, and each BL with 8 weeks outcomes of the Walking Index for Spinal Cord Injury II (WISCI II), 10-Meter Walk Test (10MWT)—self-selected speed (sss) and maximum speed (ms), six-minute walk test (6-MIN-TEST), Timed Up and Go Test (TUG) and lower extremity motor scores (LEMS) are listed chronologically. Also the mean and standard deviations of p-values of the comparison among all BL, BL with 4 weeks, and BL with 8 weeks are provided. All significant differences (p < 0.05) are marked in red.
(XLSX)

S2 Table Data on patient demographics and of all study assessments. The top row contains the descriptor of each of the assessments. The rest of the rows of the table contain the assessment data of each study participant.
(XLSX)

S3 Table Data of end user survey. The top row contains the items of the end user survey. Each of the rows of the rest of the table contains the answers of each end user to each of the items.
(XLSX)
