# Correction: Safety and Efficacy of At-Home Robotic Locomotion Therapy in Individuals with Chronic Incomplete Spinal Cord Injury: A Prospective, Pre-Post Intervention, Proof-of-Concept Study

**DOI:** 10.1371/journal.pone.0126999

**Published:** 2015-04-20

**Authors:** The PLOS ONE Staff

## Notice of Republication

This article was republished on April 10, 2015, to correct figure quality errors that were introduced during the typesetting process. The publisher apologizes for the errors. Please download this article again to view the correct version. The originally published, uncorrected article and the republished, corrected article are provided here for reference.

## Supporting Information

S1 File Originally published, uncorrected article. (PDF)

S2 File Republished corrected article. (PDF)
