# Supplementary material for: Safety and Efficacy of At-Home Robotic Locomotion Therapy in Individuals with Chronic Incomplete Spinal Cord Injury: A Prospective, Pre-Post Intervention, Proof-of-Concept Study
Source: PLoS One. 2015 Mar 24;10(3):e0119167. doi: 10.1371/journal.pone.0119167 (PMC4372343; doi:10.1371/journal.pone.0119167)
Supplement: S1 Protocol — (PDF) [file pone.0119167.s002.pdf]

STIFTUNG ORTHOPÄDISCHE UNIVERSITÄTSKLINIK HEIDELBERG

Department Orthopädie II – Rehabilitation Medicine

DIRECTOR PROF. DR. H.J. GERNER

Amendment 1 to

"MotionTherapy@Home - an automated therapy device for at-home locomotion training"

Awarded project 2005 of the „innovation competition for funding of medical technology“ of the German Federal Ministry of Education and Research (grant no. 01EZ0602)

Principal investigator:
Prof. Dr. Hans Jürgen Gerner
Phone: +49 6221 / 96 - 6322
Fax: +49 6221 / 96 - 6345

Project leader:
Dr.-Ing. Rüdiger Rupp
Phone: 06221 / 96 - 9230
Fax: 06221 / 96 - 9234

Conduction of the study
Dipl.-PW Harry Plewa

Stiftung Orthopädische Universitätsklinik
Department Orthopädie II – director Prof. Dr. H.J. Gerner
Schlierbacher Landstr. 200a
69118 Heidelberg
Germany

Date/version of the study protocol
23.07.2007 version 1.1

Date/version of the amendment
28.01.2009, amendment 1

Ethics committee application number: MV-174/2007

1 Summary

The improvement of partially or totally lost motor functions is the primary goal of the rehabilitation of patients with neurological impairments after spinal cord lesions or stroke. The restoration of the ambulatory function is the primary focus of the therapeutic efforts, because of its high relevance for reintegration into social and professional life. At the current state physiotherapeutic approaches based on motor learning principles are mainly contributing to the success of motor rehabilitation. The physiotherapeutic method investigated best in stroke survivors and individuals with incomplete spinal cord injury (SCI) is locomotion training on a motorized treadmill. Due to the fact that particularly in the very acute stage patients are not sufficiently able to initiate stepping movements voluntarily, the leg movements are supported by locomotion robots, an active tilt-table or manually by therapists. These large-scale devices are mainly used in specialized centers because of their investment and maintenance costs and need for qualified personnel for operation. With robotic devices relevant improvements in the ambulatory function of individuals with incomplete SCI were shown. However, it became apparent that sustained, long-term improvements induced by locomotion training can only be maintained if training is applied over a long period of time. Thus, it is important to have a low-cost locomotion therapy device, with which the skills achieved with large-scale devices in clinics can be maintained or even further improved in the home environment. Most recent research results show, that gait phase related, cyclic loading and stimulation of the foot sole seems to be most relevant parameter for success of the therapy.

The aim of this research project funded by the German Federal Ministry of Research and Education within its innovation award program is to develop a modular, flexible, to its technical minimum reduced concept for locomotion therapy at home. After several functional demonstrators of this concept are provided it is intended to perform a clinical pilot study in individuals with motor incomplete SCI (ASIA Impairment Scale C and D) to test the safety and efficacy of these de vices.

The study is planned as a single center, longitudinal study with assessments at baseline, therapy and follow-up intervals. Within 8 weeks of therapy the safety and efficacy of the daily application of the device will be tested. The patients will be randomly assigned to two groups, which will train with two versions of the device differing in the kind of foot sole stimulation.

For assessment of the training outcome standardized neurological scores quantifying the motor and sensory status as well as functional scores for quantification of the gait function will be applied. The assessments will be performed 4 weeks, 2 weeks and directly at therapy start (all baseline), 4 and 8 weeks after therapy start and 3 months after therapy end (follow-up).

With this project basic scientific knowledge about the possibilities for stimulation of the human spinal pattern generator will be generated and directly transferred into novel training methods and devices for automated locomotion therapy.

2 Table of contents

1 Summary 2
2 Table of contents 3
3 Introduction 4
3.1 Scientific background 4
4 Aims of the study 5
4.1 Primary outcome measures 6
4.2 Secondary outcome measures 6
5 Investigational device 6
5.1 General description 6
5.2 Effects (therapeutic, diagnostic) 8
5.3 Undesired effects, other risks, stress for study participants 8
6 Type of study 9
7 Randomization procedure 9
8 Inclusion criteria 9
9 Exclusion criteria 9
10 Study design 10
10.1 General description 10
10.2 Experimental procedure 10
10.3 Screening assessments 11
10.4 Invasive assessments 11
10.5 Non-invasive assessments 11
11 Concomitant therapies 12
12 Safety laboratory 12
13 Definition of adverse events 12
13.1 Definition of an adverse event (AE) 12
13.2 Definition of a serious adverse event (SAE) 12
13.3 Documentation and reporting 13
13.3.1 Documentation of an adverse event 13
13.3.2 Reporting and documentation of a serious adverse events 13
13.4 Procedure during and after the study 14
13.4.1 General 14
14 Criteria for abandonment from the study 14
15 Statistical design 14
16 Ethical and legal aspects 14
16.1 Ethical principles 14
16.1.1 Information for patients/study participants and informed consent 14
16.2 Legal basis: compliance with Medical Devices Act (MPG) and Federal Law on Data Protection (DSG) 14
16.2.1 Study registration 14
16.2.2 Vote of the ethical committee 15
16.2.3 Patient insurance 15
16.2.4 Data protection / review of original patient records 15
17 Signatures 15

3 Introduction

3.1 Scientific background

The improvement of partially or totally lost motor functions is the primary goal of the rehabilitation of patients with neurological impairments after spinal cord lesions or stroke. The restoration of the ability to walk is the primary focus of the therapeutic efforts, because of its high relevance for reintegration into social and professional life. Also in other patient groups such as orthopaedic or geriatric patients, who are immobilized over a substantial period of time [1], an early and intensive mobilization plays an important therapeutic and functional role. At the current state mainly physiotherapeutic approaches based on motor learning principles are contributing to the success of motor rehabilitation. The physiotherapeutic method investigated best in individuals with incomplete spinal cord injury (SCI) [2, 3, 4] and stroke survivors [5] is locomotion training on a motorized treadmill. Due to the fact that particularly in the very acute stage patients are not sufficiently able to initiate stepping movements voluntarily, these movements are supported by locomotion training robots (own work), an active tilt-table (own work) or manually by therapists. The automated locomotion therapy should be used preferably, because of the high physical workload of the therapist during manual assist, the associated limitations in the duration of the training sessions and the non-reproducible walking pattern during the training.

Most of available literature about locomotion studies reports of relevant positive adaptations on physiological as well as motor functions [6]. Dietz and colleagues [2] proved that individuals with a chronic motor incomplete SCI after several months of locomotion training improved to a degree that they were able to initiate step movements on their own. The results of a study performed by Wernig and colleagues [3] showed that a treadmill training over several weeks lead to an independent ambulation in ¾ of the study participants with incomplete SCI. Beside the improvements in motor functions also positive therapeutic effects in terms of stabilisation of the cardiovascular system (own work) and higher bone density [7] were reported.

Although body weight supported treadmill training has evolved to a standard therapy in the rehabilitation of patients with neurological gait disorders over the last two decades, its neurological and biochemical mechanisms are still not well understood. It is assumed that a reorganisation of nerval processes and structures [4] occurs. In particular, it is speculated that a spinal pattern generator (CPG) exists, which is reactivated by the task-oriented treadmill training [6, 8]. The basic functional mechanisms of this CPG neuronal network, which is fundamentally involved in the generation of cyclic movement patterns, was described on the basis of comprehensive animal experiments. While the CPG receives in the physiological condition its input from the brain, it can be reactivated in the pathological condition by repeated and intensive afferent input from the periphery. It was shown, that a physiological, gait-phase related loading of the foot sole plays a crucial role in CPG activation [9]. Moreover, the results from a couple of studies indicate that the application of mechanical vibratory stimuli represent an intensive trigger for activation of different human sensor systems [10]. However, at the current sate of technology no device exists, which combines the basic principles of gait-phase related activation of foot pressure afferents with the possibility of generating vibratory stimuli.

All established therapies that target on improvement of the ambulatory function are based on the principles of motor learning. A key principle of motor learning is that a higher number of repetitions of a movement task result in a higher therapeutic effect. Because of the trend towards shorter times of primary rehabilitation in specialized centers, a high intensity of locomotion therapy can only be achieved with an at-home training. However, currently there is no device existing for continuation of an automated robotic locomotion training at-home.

[1] Topp R.F., Ditmyer M.F., King K.F., Doherty K.F., Hornyak J.: The effect of bed rest and potential of prehabilitation on patients in the intensive care unit, AACN Clinical Issues 13 (2), 263-276, 2002

[2] Dietz V., Colombo G., Jensen L., Baumgartner : Locomotor capacity of spinal cord in paraplegic patients, Ann Neurol 37, 574-82, 1995

[3] Wernig A., Müller S., Nanassy A., Cagol E.: Laufband therapy based on ‘rules of spinal locomotion’ is effective in spinal cord injured persons, Eur J Neurosci 7, 4, 823-829, 1995

[4] Edgerton V.R., De Leon R.D., Harkema S.J., Hodgson J.A., London N., Reinkensmeyer D.J., Roy R.R., Talmadge R.J., Tillakarante N.J., Timoszyk W., Tobin A.: Topical review: Retraining the injured spinal cord, J Physiol 533 (1), 15-22, 2001
[5] Hesse S, Werner C.: Partial body weight supported treadmill training for gait recovery following stroke, Adv Neurol. 3;92:423-8, 2003
[6] Pearson K.G.: Neural Adaptation in the generation of rhythmic behaviour, Annu Rev Physiol, 62, 723–53, 2000
[7] de Bruin E.D., Frey-Rindova P., Herzog R.E., Dietz V., Dambacher M.A., Stussi E.: Changes of tibia bone properties after spinal cord injury: effects of early intervention, Arch Phys Med Rehabil.80(2), 214-20, 1999
[8] Duysens J., Van de Crommert H.W.: Neural control of locomotion; Part 1: The central pattern generator from cats to humans, Gait & Posture 7, 131-141, 1998
[9] Dietz V., Müller R., Colombo G.: Locomotor activity in spinal man: significance of afferent input from joint and load receptors, Brain 125, 2626-34, 2002
[10] Haas C.T., Turbanski S., Kaiser I., Schmidtbleicher D.: Biomechanische und physiologische Effekte mechanischer Schwingungsreize beim Menschen, Deut Zeitsch Sportmed 55 (2), 34-43, 2004

Own work

Within the last five year a couple of prototypes for locomotion therapy were developed at the Department II of the Orthopedic University Hospital Heidelberg and some clinical studies were conducted [11]. First, in cooperation with the research department of the paraplegic center of the university hospital Balgrist in Zuerich, Switzerland (Prof. Dr. V. Dietz) the prototype of a dynamic, active tilt table was developed and provided, which combines for the fist time the possibility for continuous verticalization with the generation of physiological (kinematics and kinetics) movements of the legs [12]. The safety and clinical relevance of this prototype was assessed in a pilot study with overall 8 patients with an acute motor complete SCI. The study results show that these patients with a SCI rostral to C6 and the associated loss of supraspinal sympathetic innervation of the heart can be verticalized with the active tilt table over a period of 30 minutes without any signs of a syncope or presyncope. During the therapy no decrease in blood pressure typically seen in this patient population occurred [13]. These results formed the therapeutical and legal basis for the technology transfer of the prototype into the industrially manufactured product „Erigo“ (Hocoma AG, Volketswil, Switzerland) [14].
Furthermore, our research group participated in an efficacy study of the driven gait orthosis „Lokomat“. The study results show that individuals with a chronic motor incomplete SCI, who are basically able to stand, can improve their gait speed and endurance significantly (50%) over eight weeks of “Lokomat” training [15].

[11] Rupp R., Gerner H.J.: Neue Rehabilitationstechniken beim Erwachsenen mit neuromuskulärer Erkrankung, Medizinisch-Orthopädische Technik, 124(4), 63-71, 2004
[12] Rupp R., Eberhard S., Schreier R., Colombo G.: Der Reha-Stepper - Lokomotionstherapie in der Frührehabilitation von Querschnittgelähmten, Biomedizinische Technik, Band 47, Ergänzungsband 1, 708-711, 2002
[13] Colombo G., Schreier R., Mayr A., Plewa H., Rupp R.: Novel Tilt Table with integrated robotic stepping mechanism: Design Principles and Clinical Application, IEEE Proceedings of the 9th International Conference on Rehabilitation Robotics Frontiers of the Human-Machine Interface, 227-230, 2005
[14] Dietz V., Colombo G., Rupp R.: Device and Method for Locomotion therapy, Weltweites Patent, Europäische Veröffentlichungsnummer EP11690036
[15] Wirz M., Dietz V., Zemon D.H., Rupp R., Scheel A., Colombo G., Hornby TG.: Effectiveness of an automated locomotor training in patients with a chronic incomplete Spinal Cord Injury: A Multicenter Trial, Archives for Physical Medicine and Rehabilitation, 86(4), 672-680, 2005

4 Aims of the study

The primary aim of this research project funded by the German Federal Ministry of Research and Education as a key experiment is the development and provision of a couple of prototypes of a novel robotic locomotion training device for at-home use. The aim of the clinical pilot study, which is performed in the framework of this project, is the evaluation of the safety and usability of the novel therapeutic device. Additionally, first results about the efficacy of the training should be obtained regarding to improvements of the ambulatory function of individuals with chronic motor incomplete SCI.
Standardized neurological scores for objective quantification of the integrity of the motor and sensory spinal tracts (ASIA) and assessments for the spasticity status (Modified Asworth Scale)

together with functional test focusing on walking function will be applied. The assessments will be performed 4 weeks before, 2 weeks before and directly at therapy start (all baseline), 4 and 8 weeks after therapy start and 3 months after therapy end (follow-up).
In detail, the following tests will be performed:

- ASIA motor und sensory score, ASIA Impairment Scale [17]
- Modified Ashworth Scale of the major joints of the lower extremities [18]
- WISCI II (Walking index for spinal cord injury II) [19, 20]
- 10 Meter Timed Walk Test including self-selected and maximal gait speed, Timed-up-and-go (TUG) Test [21, 22]
- 6 Minute Walk Test [23]
- SCI-FAI (Spinal cord injury functional ambulation inventory) [24]
- Münchner Lebensqualitäts-Dimensionen Liste (MLDL) [25, 26]

[17] Maynard FM Jr, Bracken MB, Creasey G, Ditunno JF Jr, Donovan WH, et al.: International Standards for Neurological and Functional Classification of Spinal Cord Injury. American Spinal Injury Association, Spinal Cord, 1997, 35(5), 266-274.
[18] Smith AW, Jamshidi M, Lo SK: Clinical measurement of muscle tone using a velocity-corrected modified Ashworth scale, Am J Phys Med Rehabil., 2002, 81(3), 202-6.
[19] Ditunno et al.: Walking Index for Spinal Cord Injury, Spinal Cord, 2000, 38, 234-243.
[20] Dittuno PL, Dittuno Jr JF: Walking index for spinal cord injury (WISCI II): scale revision, Spinal Cord, 2001, 39 (12), 654-656.
[21] Rossier P, Wade DT: Validity and reliability comparison of 4 mobility measures in patients presenting with neurologic impairment, Archives of Physical Medicine and Rehabilitation, 2001, 82(1), 9-13.
[22] Schoppen T, Boonstra A, et al.: The Timed "up and go" test: reliability and validity in persons with unilateral lower limb amputation, Arch Phys Med Rehabil 1999, 80, 825-828
[23] Harada ND, Chiu V, et al.: Mobility-related function in older adults: assessment with a 6-minute walk test, Arch Phys Med Rehabil, 1999, 80, 837-841.
[24] Field-Fote E, Fluet G, et al: The spinal cord injury functional ambulation inventory (SCI-FAI). J Rehabil Med 2001, 33, 177-181.
[25] von Steinbüchel N, Bullinger M, Kirchberger I: Die Münchner Lebensqualitäts-Dimensionen Liste (MLDL). Entwicklung und Prüfung eines Verfahrens zur krankheitsübergreifenden Erfassung der Lebensqualität. Zeitschrift für Medizinische Psychologie, 1999, 3, 99-112.
[26] Büttner A, Latarnik S, Rode S, Seiffert L, Remer H, Bennefeld H: Lebensqualität bei neurologischen Erkrankungen, Nervenheilkunde, 2005, 24 8, 675-80.

4.1 Primary outcome measures

The primary outcome measures are consisting of all tests for direct assessment of the walking function i.e., the changes of the 10 Meter Timed Walk Test, the TUG Test, the 6 Minute Walk Test and the WISCI II between baseline and end of therapy will be analyzed.

4.2 Secondary outcome measures

The secondary outcome variables consist of the motor and sensory scores of the International Standards for Neurological Classification of Spinal Cord Injury released by the ASIA (American Spinal Injury Association) for the quantification of the neurological changes and the modified Ashworth-Scale for the assessment of spasticity between baseline and therapy end.
Furthermore, the subjective evaluation of the walking function and of the quality of life with the Münchner Lebensqualitäts - Dimensionen Liste (MLDL) will be analyzed. The analysis of the data assessed at a follow-up visit 3 months after therapy end will help to answer the question, if trained skills can be maintained or will even further improve.

5 Investigational device

5.1 General description

The investigational class IIa locomotion training device consists of two active orthoses (1) for moving the knee and ankle joints, two stimulative shoes (2), an adjustable seat (3), a box with electrical and mechanical components (4) and a human-machine interface (5).

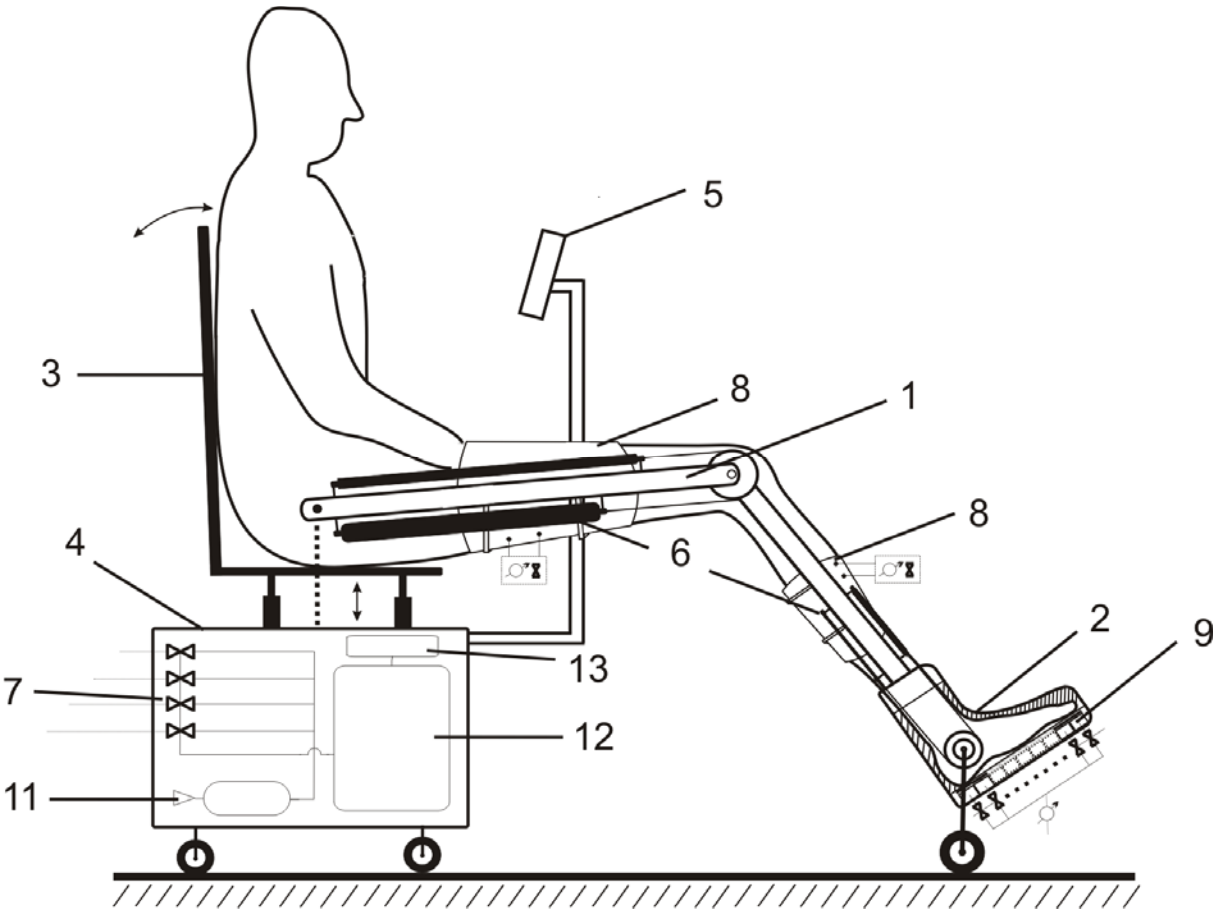

Figure 1: Overview of the main components of the novel device for locomotion training at-home

The movements of the active orthosis are generated by artificial, pneumatic muscles (6) in an antagonistic configuration. The air-mass flow of each muscle is controlled by dedicated proportional valves (7). In contrast to rigid electrical spindle drives the pneumatic muscles have the advantage of being inherently soft. This together with orthotic fittings with soft inlays (8) will ensure a safe movement of the users' legs.

The stimulative shoe generates mechanical afferent stimuli on the foot sole for activation of the spinal pattern generator. To provide the possibility for implementation of different forces and stimulation patterns, each of the mediolateral bars of the stimulative shoe can be activated separately by individual short stroke cylinders (9), which are controlled by fast switching valves.

A seat with adjustable height and inclination allows for a variation of the range of motion of the hip joint during the training.

The component box (11) mounted under the seat contains a compressor unit providing the pressurized air needed for operation of the artificial muscles. A real-time controller (12) implements a model-based, robust and safe control of the device's movements. A hardware-based, redundant watchdog (13) continuously monitors the correct operation of the real-time controller and immediately stops the operation of the device in case a malfunction is detected.

The human-machine interface visualises the training activity to the user (biofeedback), saves the measurement and training data and lets the user interactively operate the device.

<u>Main features of the MotionTherapy@Home therapy device</u>

- Movements comparable to physiological walking (can be increased up to normal gait speed of 1.0 – 1.2 m/s)
- Apparatus for user-defined configuration of the mechanical stimulation of the foot sole (in anterior-posterior direction) called stimulative shoe
- Tiltable seat backrest

- Redundant, hardware-based safety monitoring

5.2 Effects (therapeutic, diagnostic)

The primary therapeutic goal of the novel locomotion trainer is the improvement of the ambulatory function of individuals with incomplete SCI. With the continuous, intensive generation of stimuli equivalent to those of physiological walking a relevant impact on the goal-directed reorganisation of spinal as well as supraspinal neural structures is expected. Additionally, the training-induced neuroplasticity will likely result in improvements of gait speed, distance and endurance and also of gait quality (symmetry, dependency on walking aids).
Beside the direct functional effects also positive effects on the secondary consequences of SCI are expected. These more general therapeutic effects will only qualitatively be assessed. A more quantitative documentation is planned for future studies. In detail, the following positive therapeutic effects are expected:

- Antispatic effect – due to passive joint mobilisation and activation of the spinal pattern generator
- Prophylaxis of contractures– due to passive joint mobilisation with appropriate velocity
- Reduction of edema – lymph drainage due to passive muscle mobilisation and activation of the spinal pattern generator
- Stabilisation / activation of the cardiovascular system– shift of blood volume from legs into trunk due to activation of venous muscle pump
- Eupepsia – passive mobilisation of intestines and activation of the autonomous nervous system

5.3 Undesired effects, other risks, stress for study participants

- Severe osteoporosis increases the risk for fractures. In the targeted study population of individuals with motor incomplete SCI a severe osteoporosis is very unlikely because of the preserved ability to stand and carry weight on the lower extremities. Persons with a known history of osteoporosis will not be included in the study. Worldwide, to our knowledge only one incident of a fracture occurred during a therapy with the gait robot “Lokomat”, which was caused by a handling error.
- In case of improper operation of the device by the end user there is an increased risk for skin squeezing, skin abrasion or skin burns of the body regions, where the orthotic fittings support the lower extremities. To minimize these risks patients will be in detail instructed in the proper use and handling of the device.
- In principle, the device with its orthotic fittings bears the inherent risk of causing pressure sores. Although this risk is low in the included study participants with motor incomplete SCI due to their moderate to low muscular atrophy, a damage of the skin can occur particularly at the edges of the fittings. Based on the experiences with the routine application of locomotion robots in our hospital from the technical side all known measures e.g., use of soft materials for the orthotic fittings and avoidance of sharp edges, were undertaken to achieve a high level of safety and comfort for the user.
- A triggered spasticity may lead to an increased active torque of the pneumatic drives, which may result in an injury of muscles or tendons. During the training the angles of all joints are continuously monitored and the device will stop automatically in case a predefined torque threshold is exceeded. Due to the implemented drive concept based on pneumatic actuators the joint torque is inherently limited to a safe level by the maximal pressure of the compressed air.
- The other risks of the therapy correspond to those of regular physiotherapeutic interventions.

6 Type of study

The study is designed as a prospective, single center longitudinal pilot study with two therapy groups. Both groups receive therapy following the same schedule (baseline-, therapy and follow-up interval), but with two different types of foot sole stimulation (simulation physiological foot loading pattern vs. unspecific, gait-phase related vibrational stimuli). Safety and efficacy evaluation is to be made within a sample of 30 individuals with chronic, motor incomplete SCI (ASIA Impairment Scale C or D = limited ambulation possible).
As only individuals with chronic (date of injury > 1 year) SCI are included, in whom spontaneous neurological recovery does not happen, a control group is not necessary. Recruiting patients within the time after trauma, in which spontaneous recovery occurs, would drastically enlarge sample size due to this group’s high heterogeneity. Patients with spinal cord lesions due to tumors are excluded because of negative effects on walking function caused by the degenerative course of the disease.

The study design essentially matches the procedures of our study with the motor-driven gait orthosis “Lokomat” conducted 3 years ago, allowing for a direct comparison of the results of both studies.

7 Randomization procedure

Randomization procedure: The assignment to therapy group 1 (specific foot sole stimulation) and therapy group 2 (unspecific foot sole stimulation) will be randomized. The randomization will be balanced before beginning of the study into to therapy groups (0=unspecific, 1=specific) by a computer-generated list of random numbers with a block size of 2x15 patients.

Concealment: The random number list is generated before study start by the statistician of the Stiftung Orthopädische Universitätsklinik. Due to the consecutive patient recruitment a further stratification is not necessary.

Procedure: Each patient who fulfils the inclusion and exclusion criteria will be immediately reported to the statistician. The statistician tells after having being informed about the data of every consecutively included patient via phone or personally the group assignment on the randomization list in a descending order and notes the name and birth date of the patient in the list. The first patient will receive the predefined assignment of number 1, the second patient the assignment of number 2, and so on. Vice versa, the person involved in the project makes a note of the assignment. All assignments will be documented in a logbook, which is provided on request only after study end. This procedure ensures that the concealment of the group assignment defined by the rest of the randomization list is guaranteed. A drop-out patient will be replaced by the next consecutively included patient with retaining his/her group assignment.

8 Inclusion criteria

- Motor incomplete SCI (ASIA C, D)
- Time after injury > 1 year
- Age between 18 and 60 years
- SCI caused by trauma, hemorrhage/ischemia at a distinct time point or disc prolapse
- Walking Index for Spinal Cord Injury II (WISCI II) >= 5 (basically ambulatory)

9 Exclusion criteria

- Body weight > 130 kg
- Body height > 200 cm
- Leg length differences > 2 cm
- Severe contractures in hip-, knee-, and ankle joints (> 20% of normal range of motion)

- Severe osteoporosis in lower extremities
- Instable fractures, open injuries and pressure sores at torso and/or lower extremities
- Extreme spasticity
- Cardiovascular, pulmonary, metabolic or additional orthopaedic diseases or impairments restricting ambulatory function
- Psychological irregularities
- Considerable changes (> 15%) within two subsequent baseline assessments (indicator for neurological recovery)
- WISCI II > 20 and one-leg-stand (both sides) possible for at least 3 sec (= normal level of ambulation)

10 Study design

10.1 General description

Assessments are preformed 4 and 2 weeks before and directly at study onset (baseline), in the middle and at the end of the therapy interval (4 and 8 weeks after therapy onset, respectively) and 3 months after therapy completion (Follow-up). An overview is given in Figure 2:

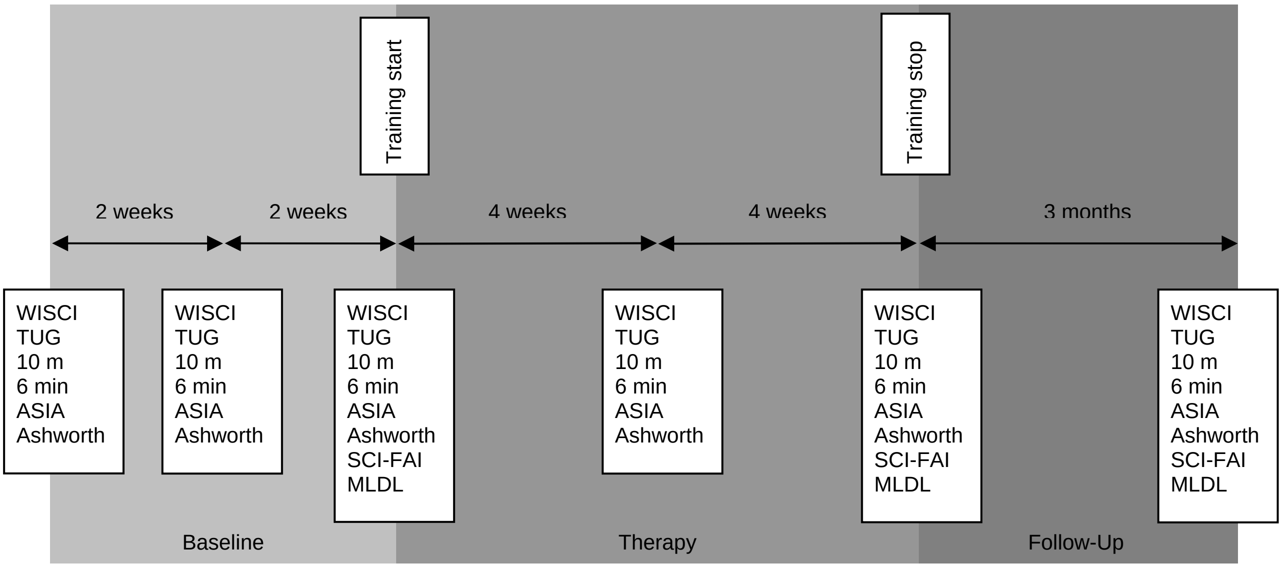

Figure 2: Overview of the study specific assessments and their schedule

The therapy period lasts 8 weeks with patients training 30-45 min. daily at least 4 and maximally 6 times per week using the novel therapy device.

10.2 Experimental procedure

- At the first therapy session patients are made familiar with the machine's functionalities and provided with a manual of instruction.
- After that, a therapist adapts the orthotic fittings, the distance between the leg orthoses, the range of motion and gait speed individually to the patient's needs. After parameters are set up correctly, a 30-min. test training is performed, allowing the patient to familiarize with the device plus providing an opportunity to the therapist to correct errors.
- After the test training the device is made available to the patient for an 8 weeks period for therapy at home. Where applicable, the device can be taken home by the patient straightaway. Otherwise it is delivered by a forwarder or personally.
- The therapy sessions will follow the following scheme:

Before a therapy session the device has to be switched on using the power switch located at the side of the seat. After this the device initiates a short self-test, checking the core components for correct functionality. On successful test conclusion, the patient transfers to the seat and fixates his legs in the designated orthotic fittings for thigh and shank, using hook and loop fasteners. The user interface allows changing the orthosis position in a way that a comfortable transfer is enabled. Finally the leg straps have to be closed concentrically over the legs and the tongue of the stimulative show needs to be manually fixated.
As soon as both limbs are safely fixed, the training can begin. To proceed with the training, a hand button has to be constantly pushed. With the hand button pushed down and the start button on the screen activated, training starts at slowest speed. The hand button enhances personal safety, as the training is instantly stopped when the button is released. The same effect is achieved when pushing the emergency button located at the side of the seating surface.

- In case the therapy is interrupted because the hand button is released accidentally, training can be resumed within 30 seconds by choosing the option „continue with training“. Otherwise, an emergency shut-down is initiated and training restart requires a complete reboot of the device (turn main power switch off and on again). Training speed can be adjusted in the user interface.
- During training, the user interface displays data which inform the patient about his active muscle force during training. For instance, a “smiley” indicates a correct active participation of the patient. The user interface also informs about elapsed training time, distance covered and device errors.
- Essential therapy data is saved on a mobile data storage device and is to be taken to study visits.

10.3 Screening assessments

During the baseline interval (4 and 2 weeks before study onset) a subset of study assessments will be performed. These are the following:

- ASIA motor and sensory score, ASIA impairment scale [17]
- Modified Ashworth-Scale [18]
- WISCI II (Walking index for spinal cord injury) [19, 20]
- 10 Meter Timed Walk Test including maximal walking speed, Timed „up and go“ (TUG) test [21, 22]
- 6 Minute Walk Test [23]

10.4 Invasive assessments

None

10.5 Non-invasive assessments

For the assessment standardized neurological scores documenting the state of neurological innervation (ASIA) and spasticity (Ashworth) are used along with functional scores quantifying walking ability.
In detail, the following clinical scores are collected:

- ASIA motor (voluntary muscle force) and sensory score (sharp-dull discrimination, light touch perception), ASIA Impairment Scale (classification of overall neurological status defined by motor and sensory scores [17])
- Modified Ashworth-Scale (assessment of joint resistance in lower extremities = categorization of spasticity) [18]
- WISCI II = walking index for spinal cord injury II (classification of dependency on walking aids) [19, 20]
- 10 Meter Timed Walk Test (time the patient needs to cover a distance of 10 m with comfortable or fast walking speed), Timed „up and go“ (TUG) test (time the patient needs to stand up, walk a distance of 6 m and sit down again) [21, 22]
- 6 Minute Walk Test (distance walked in 6 min) [23]

- SCI-FAI = spinal cord injury functional ambulation inventory (clinical estimation of gait quality) [24]
- MLDL = "Münchner Lebensqualitäts-Dimensionen Liste" (questionnaire for assessment of quality of life) [25, 26]

[17] Maynard FM Jr, Bracken MB, Creasey G, Ditunno JF Jr, Donovan WH, et al.: International Standards for Neurological and Functional Classification of Spinal Cord Injury. American Spinal Injury Association, Spinal Cord, 1997, 35(5), 266-274.

[18] Smith AW, Jamshidi M, Lo SK: Clinical measurement of muscle tone using a velocity-corrected modified Ashworth scale, Am J Phys Med Rehabil., 2002, 81(3), 202-6.

[19] Ditunno et al.: Walking Index for Spinal Cord Injury, Spinal Cord, 2000, 38, 234-243.

[20] Dittuno PL, Dittuno Jr JF: Walking index for spinal cord injury (WISCI II): scale revision, Spinal Cord, 2001, 39 (12), 654-656.

[21] Rossier P, Wade DT: Validity and reliability comparison of 4 mobility measures in patients presenting with neurologic impairment, Archives of Physical Medicine and Rehabilitation, 2001, 82(1), 9-13.

[22] Schoppen T, Boonstra A, et al.: The Timed "up and go" test: reliability and validity in persons with unilateral lower limb amputation, Arch Phys Med Rehabil 1999, 80, 825-828

[23] Harada ND, Chiu V, et al.: Mobility-related function in older adults: assessment with a 6-minute walk test, Arch Phys Med Rehabil, 1999, 80, 837-841.

[24] Field-Fote E, Fluet G, et al: The spinal cord injury functional ambulation inventory (SCI-FAI). J Rehabil Med 2001, 33, 177-181.

[25] von Steinbüchel N, Bullinger M, Kirchberger I: Die Münchner Lebensqualitäts-Dimensionen Liste (MLDL). Entwicklung und Prüfung eines Verfahrens zur krankheitsübergreifenden Erfassung der Lebensqualität. Zeitschrift für Medizinische Psychologie, 1999, 3, 99-112.

[26] Büttner A, Latarnik S, Rode S, Seiffert L, Remer H, Bennefeld H: Lebensqualität bei neurologischen Erkrankungen, Nervenheilkunde, 2005, 24 8, 675-80.

11 Concomitant therapies

Concomitant therapies are allowed to full extent.

12 Safety laboratory

There is no need for a safety laboratory in this study.

13 Definition of adverse events

An adverse event (AE) including a serious adverse event (SAE) is defined as follwos:

13.1 Definition of an adverse event (AE)

An AE is defined as any adverse and unexpected event (including e.g. abnormal laboratory findings), any symptom or disease occurring in a temporal context with the usage of a drug/medical device, independently of whether a causality is assumed or not. Above all, any change in laboratory findings or vital functions leading to termination of the respective study medication / -application or clinical trial should be regarded as AE.

13.2 Definition of a serious adverse event (SAE)

A serious adverse event is any adverse event that, independently of dose,

- leads to death.
- becomes life-threatening (danger of life must be present; potential life danger in case the event would have been more serious is not sufficient).
- causes hospitalization or extension of preexisting hospitalization.
- leads to permanent or critical disability.
- is a congenital aberration or birth defect.
- is another clinically relevant event.

13.3 Documentation and reporting

13.3.1 Documentation of an adverse event

Every adverse event is to be documented in the report form for adverse events.
In any case, the principal investigator must be clearly identifiable in this report. The report has to be signed and dated by the principal investigator. Moreover he should evaluate the causality between event and medical product.
The description of the adverse event contains the time the event occurred, its duration, severity, intensity, outcome and the relationship with the study appliance along with the treatment necessary.
The principal investigator is supposed to judge intensity of any AE according to the following classification rules:

Intensity:
- **Low:** The adverse event is transient and easy to bear by the subject/patient.
- **Medium:** The adverse event causes discomfort to the subject/patient and restricts his/her activities of daily life.
- **Severe:** The adverse event causes considerable problems to the subject/patient and his/her activities of daily life.

Consequences for therapy have to be documented, e.g. medical treatment, study exclusion or the like.

An „unexpected adverse event“, related to a medical product (drug/medical device) is defined as an adverse event whose character or severity does not go in line with the product's known data on safety and agreeableness.

Causality: In order to judge the relationship of administration of the product (drug/medical device) and the AE, the following definitions are used:

- **Certain:** A reaction showing a comprehensible temporal pattern after administration of the product (drug/medical device) or the testing of drug concentration in body tissue or –liquids, following a familiar or expected response pattern, which disappears after discontinue or reduction of medication and re-occurs at repeated exposure.

- **Probable:** A reaction showing a comprehensible temporal pattern after administration of the product (drug/medical device) and a familiar or expected response pattern to the suspicious product (drug/medical device), disappearing after discontinuing or dose reduction and not be explicable according to known clinical symptoms of the subject/patient.

- **Possible:** A reaction showing a comprehensible temporal pattern after administration of the product (drug/medical device) and a familiar or expected response pattern to the suspicious product, which can also be caused by other factors.

- **No coherence:** A reaction with enough information, so that the assumption that no relationship to the product (drug/medical device) exists can be made.

- **Judgement impossible:** A judgement of a relationship is not possible.

13.3.2 Reporting and documentation of a serious adverse events

In any case the principle investigator must be clearly identifiable in the report. The report has to be signed and dated by the principal investigator. Furthermore, it should contain an evaluation of causality between event and medical product.

13.4 Procedure during and after the study

13.4.1 General

The principal investigator monitors the course of clinical adverse events until cure or stabilization of the condition of the study participant.

14 Criteria for abandonment from the study

Individually:

- Study abandonment on patient's request
- Less than 4x30 min therapy sessions per week possible

Overall:
If no effects in the main outcome parameters can be detected in the medium-term, the whole study will be discontinued.

15 Statistical design

Normal distribution of data is checked by a Shapiro-Wilk test. For evaluation of metric variables in the presence of a normal distribution, the paired t-test is used to compare 2 assessment dates. A one factor, repeated measures ANOVA is used to compare several assessment dates.
If data is not normally distributed, statistical analysis is made using the Friedman-test. For post-hoc evaluation of the differences in walking function before, during, at the end and after training, the Wilcoxon signed-rank test is used to compare two successive assessment days.
For evaluation of the 10m-Test, 6 Min-Test and of the TUG, relative changes in performance are calculated (percentual changes relative to baseline).
Spearman's and, as the case may be, Pearson's correlation coefficients are used to compare initial locomotion capabilities in 10m-Test, 6-Min-Test and TUG and their changes (absolute and relative), as well as to correlate with ASIA, Ashworth, SCI-FAI and WISCI scores.

16 Ethical and legal aspects

16.1 Ethical principles

The study is conducted in accordance to the declaration of Helsinki (up-to-date version) considering the ICH-GCP guidelines.
Participation in the study is voluntary and can be withdrawn at any time, without a statement, and without any negative consequences on further medical care.

16.1.1 Information for patients/study participants and informed consent

Prior to study onset, subjects/patients will be informed verbally and in written form about the rationale, aim and specifically possible health benefits and risks of the intervention. Consent is documented by the participant's signature on the informed consent form.
Study data are stored over a certain period of time according to legal regulations.

16.2 Legal basis: compliance with Medical Devices Act (MPG) and Federal Law on Data Protection (DSG)

The guidelines of the German Medical Devices Act (Medizinproduktegesetz, MPG) are adhered to and the federal law on data protection (Bundesdatenschutzgesetz, BDSG) is applied. For clinical trials the according standards and regulations of ISO 14155-1:2003 (general requirements) are adhered to. For generation of the investigation specification the standard of ISO 14155-2:2003 are adhered to.

16.2.1 Study registration

According to § 20 of the German Medical Devices Act (MPG) the study is registered together with the name of the principal investigator at the federal state authority in charge.

16.2.2 Vote of the ethical committee

The study protocol is presented to the ethical committee of the medical faculty of Heidelberg university for review before study onset. No study participant/patient is included before a positive vote of the ethical committee has been received.

16.2.3 Patient insurance

According to legal provisions, an insurance for subjects/patients is issued by “Gerling Versicherung Deutschland, Von-Werth-Str. 4-14, 50670 Köln“ with insurance number 70-005989449-7.

16.2.4 Data protection / review of original patient records

The names of patients and all other confidential information are subject to legal requirements concerning confidential medical communication and data protection. Patient data are forwarded in anonymous form only. If monitoring becomes necessary during the study, it can be carried out because patients released the principal investigators from secrecy obligation in such a way that governmental health agencies may have an insight in original patient documents in order to ensure a proper study course.

17 Signatures

Principal Investigator Prof. Dr. H.J. Gerner _______ _

Project leader Dr.-Ing. R. Rupp _______ ______
