# Supplementary material for: Correction: Safety and Efficacy of At-Home Robotic Locomotion Therapy in Individuals with Chronic Incomplete Spinal Cord Injury: A Prospective, Pre-Post Intervention, Proof-of-Concept Study
Source: PLoS One. 2015 Apr 20;10(4):e0126999. doi: 10.1371/journal.pone.0126999 (PMC4404366; doi:10.1371/journal.pone.0126999)
Supplement: S1 File — (PDF) [file pone.0126999.s001.pdf]

PLOS | ONE

RESEARCH ARTICLE

Safety and Efficacy of At-Home Robotic Locomotion Therapy in Individuals with Chronic Incomplete Spinal Cord Injury: A Prospective, Pre-Post Intervention, Proof-of-Concept Study

Rüdiger Rupp[1]*, Daniel Schließmann[1], Harry Plewa[1], Christian Schuld[1], Hans Jürgen Gerner[1], Norbert Weidner[1], Eberhard P. Hofer[2], Markus Knestel[2]

1 Heidelberg University Hospital, Spinal Cord Injury Center, Heidelberg, Germany, 2 Ulm University, Institute of Measurement, Control and Microtechnology, Ulm, Germany

* Ruediger.Rupp@med.uni-heidelberg.de

CrossMark
click for updates

OPEN ACCESS

Citation: Rupp R, Schließmann D, Plewa H, Schuld C, Gerner HJ, Weidner N, et al. (2015) Safety and Efficacy of At-Home Robotic Locomotion Therapy in Individuals with Chronic Incomplete Spinal Cord Injury: A Prospective, Pre-Post Intervention, Proof-of-Concept Study. PLoS ONE 10(3): e0119167. doi:10.1371/journal.pone.0119167

Academic Editor: Felix Schlachetzki, University of Regensburg, GERMANY

Received: April 12, 2014

Accepted: January 12, 2015

Published: March 24, 2015

Copyright: © 2015 Rupp et al. This is an open access article distributed under the terms of the Creative Commons Attribution License, which permits unrestricted use, distribution, and reproduction in any medium, provided the original author and source are credited.

Funding: This project was supported by the German Federal Ministry of Education and Research (BMBF, www.bmbf.de) under grant no. 01EZ601 and 01EZ0602 from 2006-2010 and from 2011-2014 by the German Federal Ministry of Economic affairs and Energy (BMWi, www.bmwi.de) on the basis of a decision by the German Bundestag under grant no. KF2906701. Additionally, financial support was received by the Deutsche Forschungsgemeinschaft and Ruprecht-Karls-University Heidelberg within the funding program "Open Access Publishing". The

Abstract

Background

The compact **M**otorized orthosis for h**o**me **re**habilitation of **Gait** (MoreGait) was developed for continuation of locomotion training at home. MoreGait generates afferent stimuli of walking with the user in a semi-supine position and provides feedback about deviations from the reference walking pattern.

Objective

Prospective, pre-post intervention, proof-of-concept study to test the feasibility of an unsupervised home-based application of five MoreGait prototypes in subjects with incomplete spinal cord injury (iSCI).

Methods

Twenty-five (5 tetraplegia, 20 paraplegia) participants with chronic (mean time since injury: 5.8 ± 5.4 (standard deviation, SD) years) sensorimotor iSCI (7 ASIA Impairment Scale (AIS) C, 18 AIS D; Walking Index for Spinal Cord Injury (WISCI II): Interquartile range 9 to 16) completed the training (45 minutes / day, at least 4 days / week, 8 weeks). Baseline status was documented 4 and 2 weeks before and at training onset. Training effects were assessed after 4 and 8 weeks of therapy.

Results

After therapy, 9 of 25 study participants improved with respect to the dependency on walking aids assessed by the WISCI II. For all individuals, the short-distance walking velocity measured by the 10-Meter Walk Test showed significant improvements compared to

funders had no role in study design, data collection and analysis, decision to publish, or preparation of the manuscript.

Competing Interests: D. Schliessmann, C. Schuld, H. J. Gerner, N. Weidner declare no potential conflicts of interest with respect to the research, authorship, and/or publication of this article. Three co-authors, R. Rupp, E. P. Hofer and M. Knestel are holders of a European patent (No. EP000001959909A1) for the MoreGait device, does not alter the authors' adherence to PLOS ONE policies on sharing data and materials. The authors confirm that the fact that one of the co-authors, N. Weidner, serves as an editor for PLOS ONE does not alter the authors' adherence to PLOS ONE editorial policies and criteria.

baseline (100%) for both self-selected (Mean 139.4% ± 35.5% (SD)) and maximum (Mean 143.1% ± 40.6% (SD)) speed conditions as well as the endurance estimated with the six-minute walk test (Mean 166.6% ± 72.1% (SD)). One device-related adverse event (pressure sore on the big toe) occurred in over 800 training sessions.

Conclusions

Home-based robotic locomotion training with MoreGait is feasible and safe. The magnitude of functional improvements achieved by MoreGait in individuals with iSCI is well within the range of complex locomotion robots used in hospitals. Thus, unsupervised MoreGait training potentially represents an option to prolong effective training aiming at recovery of locomotor function beyond in-patient rehabilitation.

Trial Registration

German Clinical Trials Register (DKRS) DRKS00005587

Introduction

Loss of mobility has devastating effects on the quality of life of those affected and their ability to remain independent in the community. This applies to individuals with lesions of the central nervous system (CNS) sustained for example through stroke or spinal cord injury (SCI). In subjects with incomplete SCI (iSCI), intensive gait training leads to substantial improvements in walking function [1, 2]. The fundamental concept of motor function restoration is based on the notion that repeated execution of motor tasks induces plasticity—functional and structural reorganization of neuronal circuits—in the injured brain and spinal cord [3, 4]. A neuroanatomical structure that is particularly relevant for locomotion is the central pattern generator (CPG), which consists of a cluster of neuronal networks in the spinal cord [5–8] and is involved in the generation of stepping-like movements in supine and upright position in humans [9, 10]. In completely spinalized animals, the CPG can be trained by means of appropriate paradigms [11]. A series of recently conducted animal experiments introducing a chronological spinal cord dual-lesion paradigm revealed that in incomplete spinal lesions, adaptations of the CPG contribute to a larger extent to the recovery and re-expression of the locomotor pattern than was previously assumed [12]. To achieve a relevant level of independent locomotion, the CPG needs sufficient supraspinal input, [13] as well as afferent feedback from the peripheral nervous system [14].

Several factors of motor learning—task specificity, repetition, active participation and appropriate intrinsic and extrinsic feedback—have been identified as contributing to the long-term retention of a newly acquired skill [15, 16]. One clinical concept that successfully capitalizes on these principles is body weight-supported treadmill training (BWSTT) [17–19], which has been further transformed from manually assisted to automated BWSTT, including motor-driven gait orthosis [20] or specialized locomotion training devices [21]. While feasibility of robot-based locomotor training has been shown, its superiority to conventional gait training still has to be demonstrated [22]. Automated treadmill training substantially reduces therapists' physical workload while also allowing the number of task-specific repetitions to be increased [23]. High-frequency task-oriented gait training requires substantial technical and personal support, which can primarily be provided by in-patient rehabilitation facilities. However,

increasing economic constraints in health care require the duration of SCI in-patient rehabilitative treatment to be shortened [24]. Consequently, with earlier transition into out-patient and home-based therapy, the quantity and quality of rehabilitation training is dramatically reduced. Moreover, comparable studies in individuals with chronic iSCI suggest that long-term, mid-intensity locomotion training over a period of several months is more effective than application of high-intensity training protocols for only a few weeks [25, 26]. These facts underline the need to continue intensive locomotor training beyond in-patient rehabilitation.

Patients can most easily incorporate practice in their daily lives in a home-based training regimen. This may offer the advantage of practice within their personal space, where problem-solving is highly motivated [22]. Locomotion robotic systems may effectively support home-based training approaches by ensuring the generation of physiological movements and by providing objective feedback on training results. While the need for technically advanced locomotion therapy systems for home use is obvious, to date no such systems are available. A simple transfer of the existing devices to the patients' homes is not possible, since most of them are restricted to application in a clinical or out-patient setting due to their size, weight and price. In addition, all of the devices have to be operated by skilled therapists. The main technical challenges of a home-based locomotion therapy device involve safety issues and its operation by the users themselves.

The **M**otorized orthosis for h**o**me **re**habilitation of **Gait** (MoreGait) is a robotic locomotion training device which has been developed and specifically designed for unsupervised, home-based therapy [27]. From a neurobiological point of view, it aims to promote neuroplasticity at different levels within the CNS: 1) It aims to generate important sensory stimuli, which were found to activate the CPG at the spinal level [14, 28, 29] and 2) it provides external feedback about the patient's movement performance aiming at the compensation of the loss of sensation and/or proprioception and the enhancement of motor relearning at a supraspinal level [30]. From 2006 to 2008, 5 prototypes were built exclusively for research purposes and tested in several end users with iSCI [31].

The aim of this prospective, pre-post intervention proof-of-concept study was to test the safety of autonomous locomotor training with the MoreGait prototypes at the homes of individuals with sensorimotor iSCI and to obtain preliminary data about its efficacy. The pilot study results indicate that home-based robotic locomotion training with MoreGait is feasible and safe. The magnitude of functional improvements achieved by MoreGait training in individuals with iSCI is well within the range of complex locomotion robots used in hospitals.

Study Participants and Methods

Participants

For this prospective, baseline-controlled, single center cohort proof-of-concept study, inclusion criteria were (1) age between 18 and 60, (2) chronic (at least 1 year after trauma), (3) traumatic or ischemic/haemorrhagic sensorimotor iSCI (ASIA Impairment Scale (AIS) C, D [32]) and (4) with at least limited household ambulation (Walking Index for Spinal Cord Injury II [33] (WISCI II) > = 5). Exclusion criteria were body weight over 130 kg, height over 200 cm, contractures restricting the range of motion (ROM) to less than 80% of normal ROM, extreme spasticity, pressure sores, severe osteoporosis as well as any disease condition other than iSCI interfering with walking ability. Dropout criteria were the participant's request to withdraw from the study and a weekly therapy intensity less than 4x 30 minutes. Study participants were identified by screening of the institutional database or were informed by advertisement on the institutional and other webpages. Additionally, a call for study participation was published in a magazine for people with disabilities focusing on individuals with SCI [34].

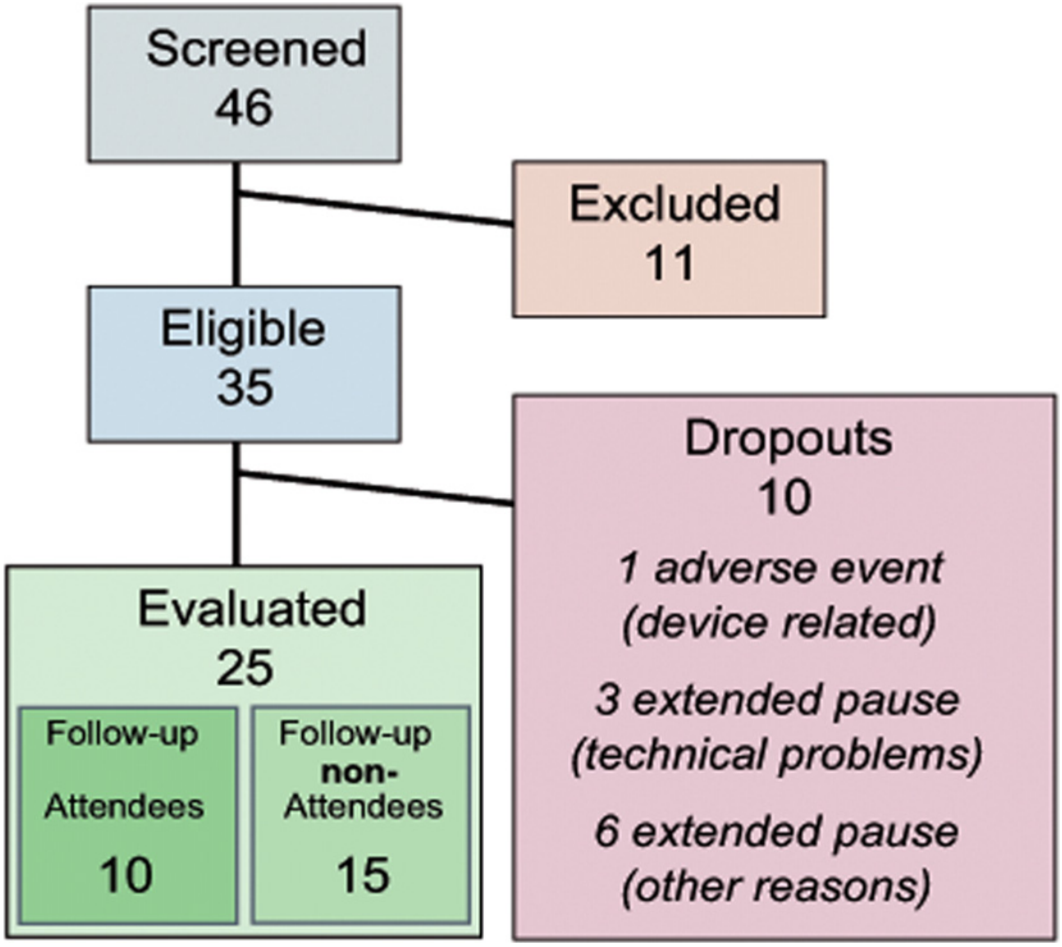

Fig 1. CONSORT flow diagram. The numbers of subjects involved in the different phases of the study are shown in the diagram.

doi:10.1371/journal.pone.0119167.g001

Between January 2009 and January 2011, 46 individuals were screened, from whom 35 were included in the study. The intended number of study participants finalizing the training was set to 30 prior to the start of study due to the limited number of available prototypes and due to funding and time constraints. Twenty-five individuals (11 female, 14 male; 5 tetraplegic, 20 paraplegic; mean age: 44.0 ± 12.4 (SD) years) with chronic (mean time since injury: 5.8 ± 5.4 (SD) years) sensorimotor iSCI (7 AIS C, 18 AIS D; WISCI II from 5 to 19) completed the training procedure (Fig. 1). Ten of these twenty-five individuals who completed the training took part in the follow-up assessment.

Ethics statement

The study was approved by the Ethics Committee of Heidelberg University Hospital (vote no. MV-174/2007) and was conducted according to the World Medical Association Declaration of Helsinki and the Guidelines for Good Clinical Practice. The protocol submitted to the ethical committee for this clinical trial and supporting TREND checklist are available as supporting information (see S1 TREND Checklist and S1 Protocol).

It has been registered at the German Institute for Medical Documentation and Information (DIMDI) as a clinical trial (registration no. 9053) with a novel medical product according to the guidelines of the European Medicinal Devices Act and with the main ID DRKS00005587 in

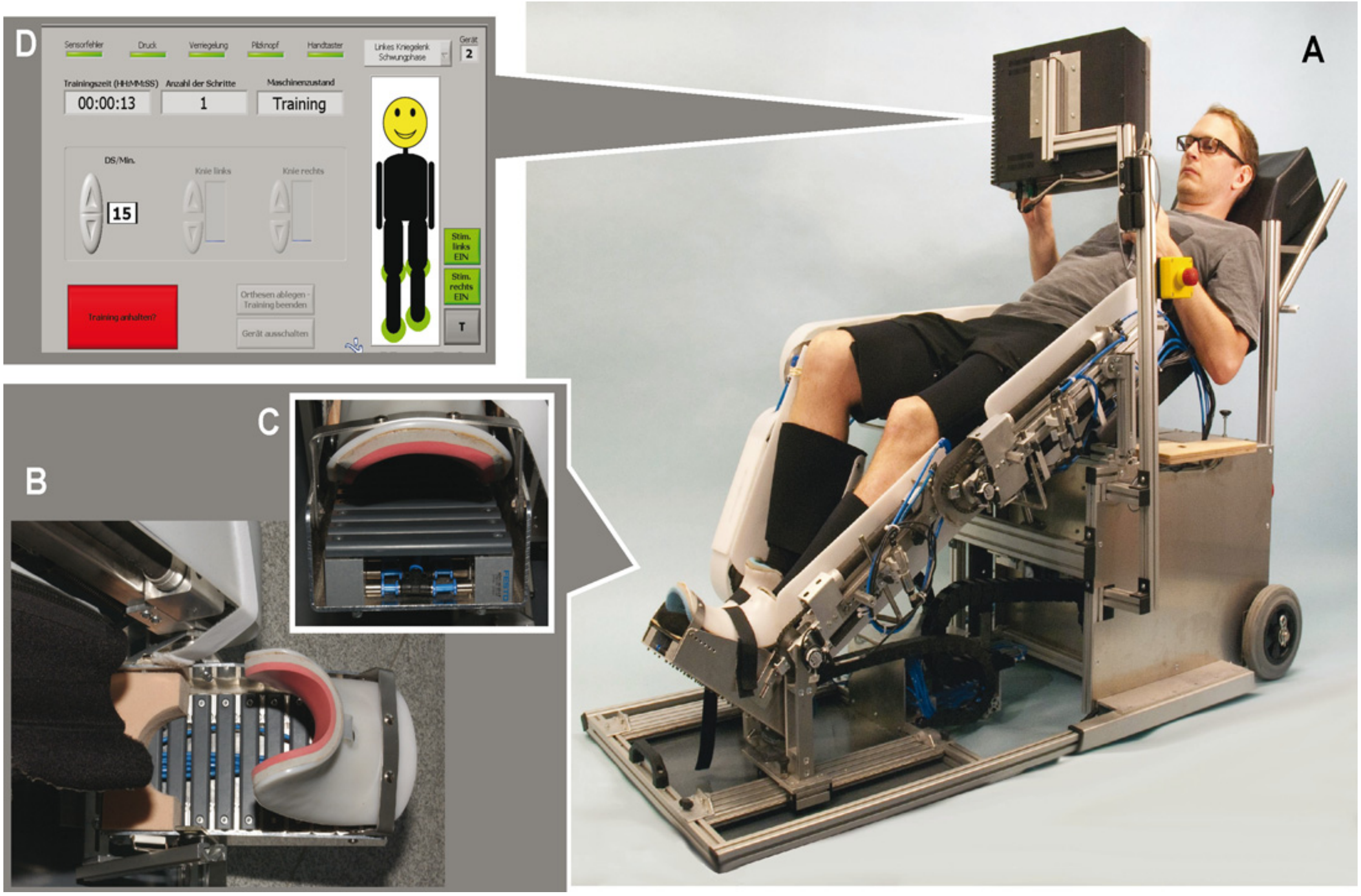

Fig 2. Overview of the components of the MoreGait device. A subject during training in the MoreGait device (A), top (B) and front (C) view of the mediolateral bars of the stimulative shoe, user interface and feedback screen (D).

doi:10.1371/journal.pone.0119167.g002

the the German Clinical Trials Register (DKRS). Participants gave written informed consent prior to study inclusion. The individual shown in Fig. 2 of this manuscript has given written informed consent (as outlined in PLOS consent form) to publish his case details.

Robotic device

The MoreGait device used in this study consists of a special seat combined with an inclined backrest, a pneumatically driven gait orthosis for each side to assist movements of both legs in the sagittal plane (independently driven knee and ankle joint, hip joint mechanically linked to knee joint via a fixed kinematic chain that allows only horizontal movements of the ankle joint) and a dedicated mechanical foot stimulation unit. Its dimensions are 172 x 70 x 130 cm (l x w x h) and the total weight is approximately 115 kg (Fig. 2A).

Pneumatic fluidic muscles (Festo AG & Co. KG, Esslingen, Germany) were selected as actuators. They offer the advantage of inherent low stiffness, which results in soft, safe and comfortable movements. Additionally, control parameters were set to allow for deviations of up to 5° from the predefined movement trajectory [35, 36], which permits the user to explore different muscular activation strategies to follow the reference trajectories.

For safety reasons, the user's body is placed in a semi-supine position. In this configuration, sufficient loading of the foot sole during stance phase cannot be generated by the user's own body

weight. Therefore a novel device—a "stimulative shoe"—was developed to mimic the loading of the foot sole without requiring the patient to be completely verticalized. This mechanical stimulation unit consists of 10 mediolateral plastic bars, which are mounted on pairs of pneumatically driven short-stroke cylinders (Fig. 2B and Fig. 2C). The generated foot loading reaches approx. 30% body weight. The timing of actuation with a sequential stance-phase related cylinder activation sequence starting at the heel and ending in the toe region and the force of each pair of cylinders can be set by software, which forms the basis for generating a physiological loading pattern. The stimulative shoe is by design also capable of generating artificial loading patterns such as gait-phase related vibrational stimuli. However, in its current version the mobile compressor does not provide enough air-mass per minute to apply sufficient force in vibration mode. For this reason, the initial plan to randomly assign the study participants into two groups, who receive a therapy either based on physiological or vibrational loading of the foot sole, was abandoned.

One of the key factors for any kind of locomotion therapy to succeed is active participation by the patients. In order to continuously provide the users with information about whether they are performing training correctly, a feedback functionality was implemented. A rating measure is calculated from the user's estimated active torques, and both the progress of the training and the absolute performance level are visualized on a display (Fig. 2D).

The right orthosis as well as the backrest can be lowered manually, enabling the patient to transfer autonomously to the lowered backrest. After successful transfer, the backrest can be inclined and the orthosis can be lifted in the training position. Leaving the device is performed in reverse order.

Study protocol

After screening and study inclusion, 3 assessment visits(0, 2 and 4 weeks) within a 4-week baseline period were planned prior to training onset, followed by assessments in the middle and at the end of an 8-week training period and a follow-up assessment 3 months after the end of training (Fig. 3) [37]. Assessments were performed by unblinded examiners either at the Spinal Cord Injury Center of Heidelberg University Hospital or at the study participants' homes. Baseline assessments and follow-up assessments were done at the Spinal Cord Injury Center, whereas the majority of 4-week and 8-week assessments were conducted at the participants' homes.

The assessments of each visit consisted of a set of well-established functional and neurological tests. The assessment scheme and outcome parameter were defined prospectively before the start of the study and was not changed over the course of the study. The self-selected WISCI II was defined as the primary outcome measure of the study due to its importance for the participants [38]. The WISCI II is a 21-item ordinal scale that classifies dependency on walking aids over a distance of 10 meters (0 = not able to walk 10meters, 20 = walking without help for 10 meters). For quantification of walking ability, the 10-Meter Walk Test (10MWT) at self-selected speed (10MWT(sss)) as well as maximum speed (10MWT(ms)), the Timed Up and Go Test (TUG) and the six-minute walk test (6-MIN-TEST) were applied [39, 40]. The 10MWT measures the time required to walk 10 meters and quantifies the short-duration walking speed. The TUG measures the time required for a patient to stand up from an armchair, walk 3 meters, return to the chair, and sit down. The 6-MIN-TEST measures the distance walked within 6 minutes and serves as a measure for endurance. All walking tests were performed with the participants using their currently appropriate walking aid(s) on even and straight walkways. If tests were performed at the participant's home, walking tests were done on barrier free, even grounds, like sports fields or non-crowded roads.

Dermatomes and myotomes as defined by the International Standards for Neurological Classification of Spinal Cord Injury (ISNCSCI) [41] were examined to check for spontaneous

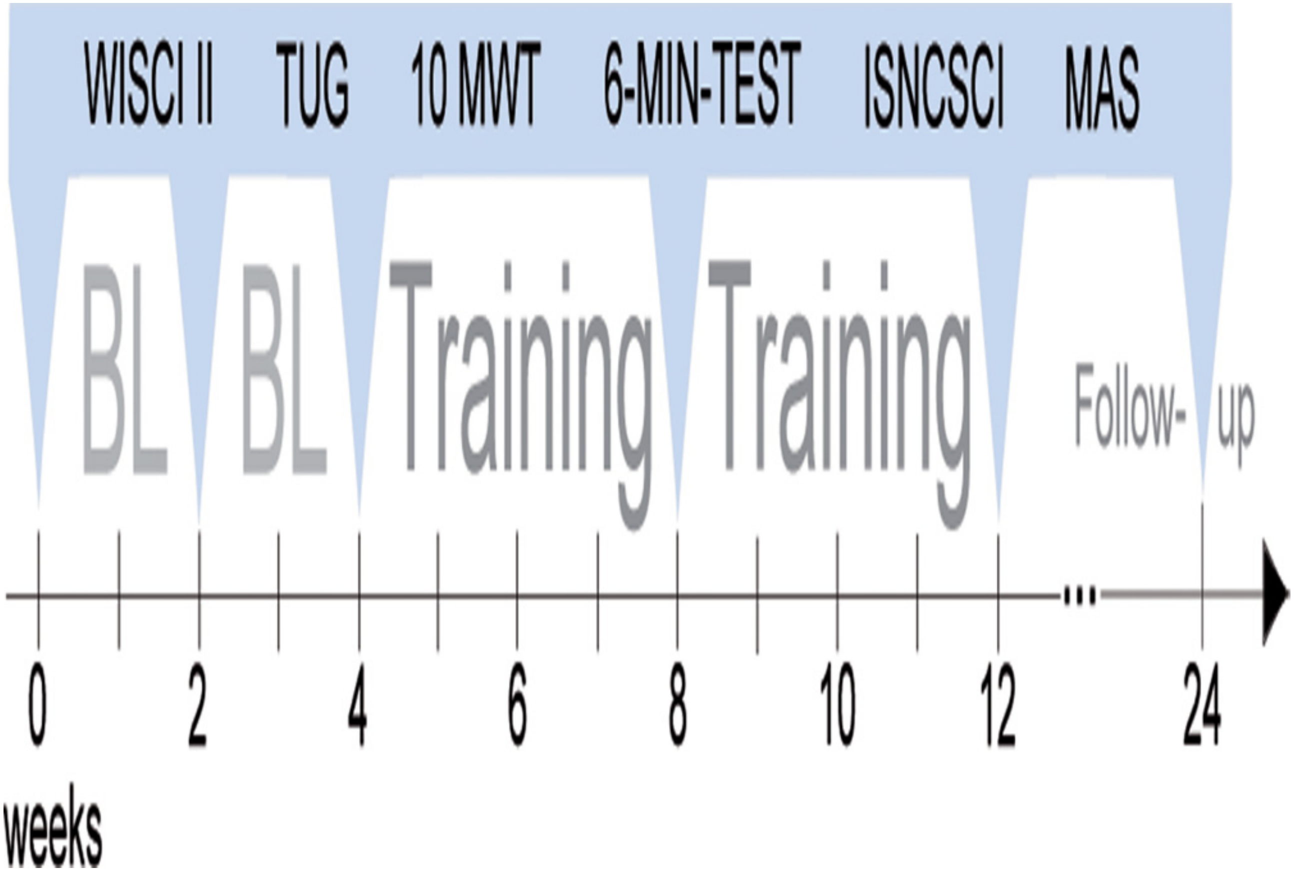

Fig 3. Study protocol showing the course of the study and its assessments on a timeline. Three baseline (BL) assessments within the first 4 weeks are followed by 2 assessments at the middle and end of the training period. The follow-up assessment was carried out 3 months after the end of training. Assessments included the Walking Index for Spinal Cord Injury II (WISCI II), Timed Up and Go Test (TUG), 10-MeterWalk Test (10MWT) at both self-selected and maximum speed, six-minute walk test (6-MIN-TEST), as well as International Standards for Neurological Classification of Spinal Cord Injury (ISNCSCI) assessments and measurement of spasticity according to the modified Ashworth scale (MAS).

doi:10.1371/journal.pone.0119167.g003

neurological recovery during baseline. ISNCSCI's lower extremity motor score (LEMS) was used to specifically assess the changes in strengths of the key muscles of the legs during the training period. A high-quality ISNCSCI assessment was assured by trained assessors [42] and computer-based scaling, scoring and classification of the ISNCSCI [43]. To determine the degree of spasticity in hip, knee and ankle joints in terms of increased resistance against passive movements in the sagittal plane, the modified Ashworth scale (MAS) was administered [44].

Training

During the 8-week therapy period, individuals trained with the MoreGait device for 30–45 minutes per day, 4 to 6 days per week. Users were instructed to set step frequency at a comfortable level to avoid fatigue during each session. The training took place at the participants' homes without supervision of the study personnel (mean distance from the spinal cord injury center in Heidelberg 209.12 ± 162.16 km; Google Maps (http://maps.google.com), fastest route). Prior to the first training session, each participant was familiarized with proper usage of the MoreGait machine. The 30-minute instruction included practicing the transfer procedure for getting into and out of the device and securing the thigh and shank straps to the legs, along with explaining the elements of the user interface displayed on the touch screen together with

the correct selection of therapy parameters. During a short trial session, an experienced technician or scientist adjusted the length of the machine's linkages and position of the orthotic fittings in order to ensure correct alignment of technical and anatomical joints. To help isolate the effect of the training device, participants were instructed not to modify their regular physical therapy, unsupervised training program, or antispastic medication during the study period.

User survey

To assess the users' satisfaction with technical design, safety and therapeutic functionality of the MoreGait device, a user survey was made. The survey consisted of a paper questionnaire which was sent to each study participant who finished the 8-weeks of therapy. The proprietary questionnaire consisting of 51 questions was designed for obtaining dedicated user feedback on details of the MoreGait device. It mainly used a five-point scale for rating of answers, which is known from other standardized surveys on assistive technology [45], supplemented by Yes/No questions and comment fields.

Twenty of the twenty-five study participants, who completed the 8-weeks MoreGait training, replied to the survey. The results were grouped into the three main categories "Therapy outcome", "Transfers, fastening and release" and "Training experience" including perception of safety. Results were analyzed on a descriptive basis eg, with boxplots.

Statistical analyses

Statistical analysis was performed with R 2.15.1 [46]. Friedman's test was used to test for overall significance of differences between all assessments, including baseline (BL1, BL2, BL3) assessments. For statistical analysis, raw values were used. Significance level was set to $\alpha = 0.05$. Pairwise Wilcoxon-Mann-Whitney tests were used for post hoc comparisons between all assessment visits, including baseline visits. To address the multiple testing problem, Benjamini-Hochberg-corrections [47] were applied. The 4-week and 8-week assessments were compared separately with each of the three baseline assessments. Differences to baseline were considered significant, if the mean of the 3 p-values from the comparison of each baseline assessment with the respective therapy assessment was $\bar{P} < 0.05$. An overview of all p-values and confidence intervals is provided in S1 Table. As non-parametric tests were used, confidence intervals (ci) are intervals of the differences of the location parameters.

Baseline stability was tested in the context of the Friedman's test. The baseline is considered stable, if pairwise post hoc tests of all three baseline assessments are not significant.

If not stated otherwise, numbers are displayed as mean ± SD. To allow for a more generalized analysis of the assessments of different study participants at 4 and 8 weeks of therapy, percentage values are calculated which are normalized to the mean baseline representing 100%.

The follow-up analysis was reduced to descriptive statistics because of the low number of available datasets for this stage (N = 10). Data from individuals that did not complete the study were not included in the analysis.

Results

Outcomes on safety and dropouts

One device-related adverse event (Grade 2–3 [48] pressure sore on the tip of the left big toe) caused 1 participant to drop out. The pressure sore was caused by a pressurized bar of the stimulative shoe, which applied its full load to the tip of the big toe. It healed after discontinuation of therapy and wound dressing for 2 weeks. After implementation of a foot length-dependent deactivation of most anterior mediolateral bars of the stimulative shoe, no further adverse

events occurred. Other reasons for dropouts were prolonged periods of non-use, such as those resulting from bladder infections or technical problems in combination with delayed repair due to great distances between the users' homes and the authors' institutions (Fig. 1). Individuals who did not attend the follow-up assessment, but completed the training were not considered as dropouts.

Stability of baseline

Baseline stability was shown for all outcome measures, since all baseline post hoc comparisons were not significant (see S1 Table for detailed information). During baseline, three temporary conversions occurred (AIS C to B and back to C, AIS C to D and back to C, and AIS D to C and back to D).

Outcome measures during therapy

A numerical overview of the absolute outcome measures is given in Table 1, while Fig. 4 presents the course of the changes in the walking test results relative to baseline as a graph.

The primary outcome measure, WISCI II, showed overall significant results ($p < 0.0006$) and increased from 12 (9 to 16) to 16 (15 to 16) (baseline to 8 weeks therapy; median

Table 1. Numerical summary of outcome measures from 6 visits.

Outcome measure \ Visit	Baseline 1	Baseline 2	Baseline 3	Mean / Median Baseline*	4 weeks therapy	8 weeks therapy	8 weeks therapy[b], *N* = *10*	Follow-up(20 weeks)[b], *N* = *10*
WISCI II [score][a]	12 (9–16)	12 (9–16)	12 (9–16)	12 (9–16)	16 (11.75–16); {24}	16 (15–16); {24}	16 (12–18.25)	14 (11.25–16)
MAS [score][a]	2 (0–9)	1 (0–4)	2 (0–4.5); {24}	1 (0–6.33)	0 (0–6)	1 (0–4.5); {24}	0 (0–8.25)	1 (0–7.0)
10MWT (sss) [m/s]	0.37 ± 0.27	0.36 ± 0.24	0.37 ± 0.24	0.37 ± 0.25	0.42 ± 0.30;{24}	0.47 ± 0.27;{24}	0.52 ± 0.35	0.59 ± 0.42
10MWT (ms) [m/s]	0.47 ± 0.43	0.45 ± 0.35	0.46 ± 0.37	0.46 ± 0.38	0.52 ± 0.42; {24}	0.57 ± 0.35; {24}	0.66 ± 0.46	0.71 ± 0.49
6-MIN-TEST [m]	117.04 ± 103.87; {24}	115.63 ± 103.08; {24}	119.46 ± 109.78; {24}	117.38 ± 104.59	142.74 ± 107.01; {23}	164.74 ± 115.03; {23}	185.1 ± 154.88	210.70 ± 162.96
TUG [s]	67.71 ± 57.89; {24}	61.60 ± 51.62	56.18 ± 38.34	61.34 ± 48.02	50.83 ± 36.10; {24}	37.21 ± 22.71; {24}	37 ± 25.36	50.80 ± 54.96
LEMS [points]	30.32 ± 9.33	29.72 ± 8.99	29.92 ± 9.24	29.99 ± 8.97	33.36 ± 10.87	36.42 ± 9.77; {24}	35.1 ± 11.28	33.70 ± 11.49
MS L2 [points]	7.48 ± 1.05	7.32 ± 1.35	6.84 ± 1.46	7.21 ± 1.13	7.76 ± 0.93	7.88 ± 1.20	8.1 ± 1.37	8.20 ± 1.69
MS L3 [points]	7.60 ± 1.32	7.36 ± 1.70	7.48 ± 1.42	7.48 ± 1.24	8.40 ± 1.58	8.52 ± 1.56	9.1 ± 0.99	8.60 ± 1.51
MS L4 [points]	4.84 ± 3.21	4.68 ± 3.22	5.04 ± 3.36	4.85 ± 3.14	5.80 ± 3.56	6.48 ± 3.33	6.7 ± 3.30	6.00 ± 3.37
MS L5 [points]	5.04 ± 3.61	4.96 ± 3.48	5.00 ± 3.42	5.00 ± 3.44	5.44 ± 3.63	6.08 ± 3.98	5.1 ± 4.58	5.00 ± 4.55
MS S1 [points]	5.16 ± 3.22	5.32 ± 3.18	5.60 ± 3.11	5.36 ± 3.02	6.24 ± 3.46	6.80 ± 3.34	6.1 ± 3.87	5.90 ± 4.01

Mean ± standard deviation of Walking Index for Spinal Cord Injury II (WISCI II), modified Ashworth scale (MAS), 10-Meter Walk Test (10MWT)—self-selected speed (sss) and maximum speed (ms), six-minute walk test (6-MIN-TEST), Timed Up and Go Test (TUG) and lower extremity motor scores (LEMS) are listed chronologically. Segmental motor scores (MS) for myotomes L2—S1 are also provided. Sample sizes are displayed in "{}" where they deviate from N = 25.

[a]Median and 25%-75% quartiles are given for ordinal scales eg, WISCI II and MAS.

[b]As follow-up data have a sample size of N = 10 the corresponding subset of patients within the 8-week therapy assessment is presented for better comparison.

doi:10.1371/journal.pone.0119167.t001

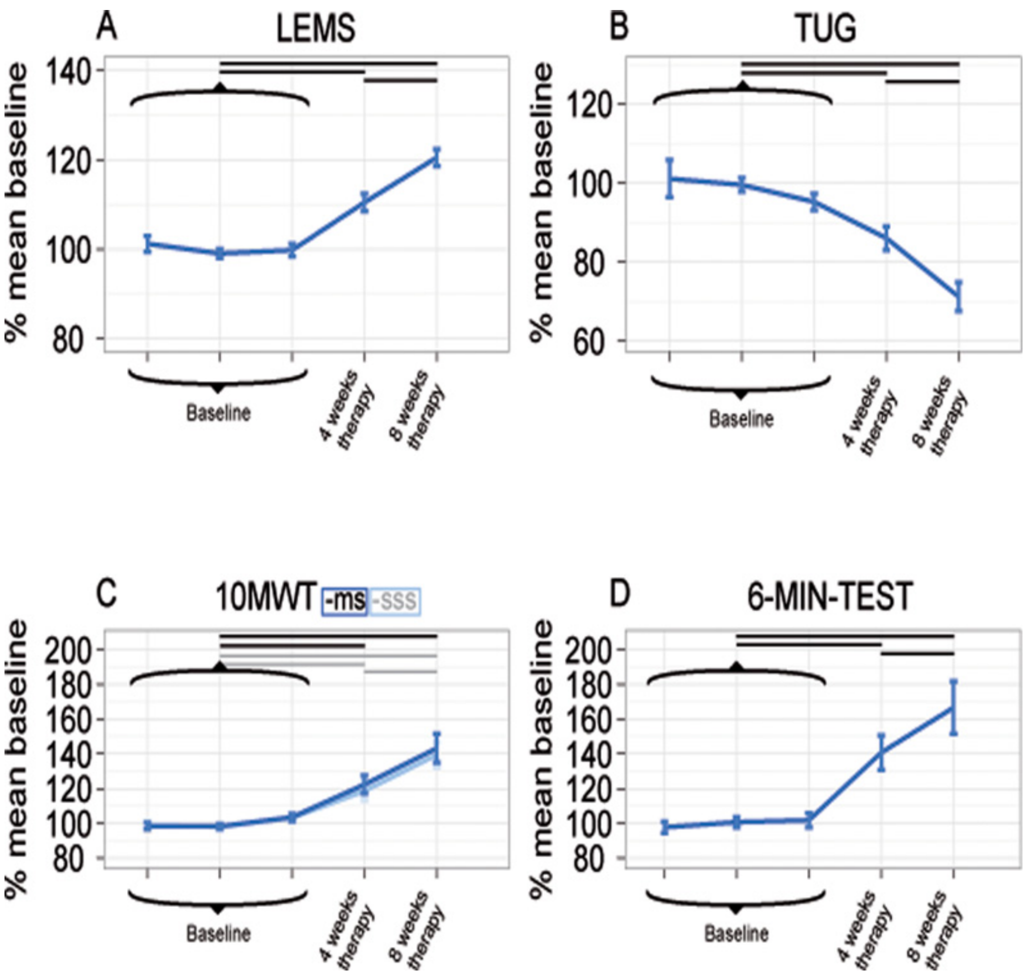

Fig 4. Overview of all study visits in relation to mean baseline. Percent of the mean baseline ± standard error (SE) along the course of the study in relation to the mean baseline for (A) Lower extremity motor score (LEMS), (B) Timed Up and Go Test (TUG), (C) 10-MeterWalk Test (10MWT) at maximum speed (-ms) and self-selected speed (-sss), (D) six-minute walk test (6-MIN-TEST). Horizontal bars mark significant differences ($p < 0.05$), which were determined on the basis of the absolute values in Table 1.

doi:10.1371/journal.pone.0119167.g004

(interquartile range (IQR))). Nine participants (2/7 AIS C, 7/18 AIS D) were less dependent on walking aids after therapy than before. A qualitative analysis of the AIS subgroups revealed that subjects classified as AIS C show a trend towards a higher improvement than those classified as AIS D (median of increase from baseline to end of therapy in AIS C = 7 and in AIS D = 4). Post hoc tests in WISCI II revealed a significantly ($\bar{P} = 0.0352, \pm 0.0035$) higher WISCI II level after 8 weeks of training compared to baseline, with a positive tendency ($\bar{P} = 0.1470, \pm 0.0504$) already identified after 4 weeks.

The results of the 10MWT for both self-selected (139.4% ± 35.5%; 8-weeks assessment) and maximum (143.1% ± 40.6%; 8-weeks assessment) speed conditions showed a significant overall improvement (both $p < 0.0001$) in short-distance walking velocity. Post hoc analysis revealed a significant increase of self-selected ($\bar{P} = 0.0015 \pm 0.0006$) and maximum ($\bar{P} = 0.0042, \pm 0.0054$) walking speed between baseline and end of therapy. Moderate improvements are already visible between baseline and 4-weeks assessments ($\bar{P} = 0.0317 \pm 0.0156$ for self-selected speed and $\bar{P} = 0.0497 \pm 0.0269$ for maximum speed). Moreover, self-selected speed increased significantly ($p = 0.0227$) between week 4 and 8. Endurance estimated with the 6-MIN-TEST

(overall significance $p < 0.0001$) showed significant improvements in post hoc tests at the end of therapy (166.6% ± 72.1%, $\bar{P} = 0.0006 \pm 0.0004$), as well as during the first half ($\bar{P} = 0.0019 \pm 0.0006$) and the second half of the therapy period (week 4 to week 8, $p = 0.0026$). Outcomes for the TUG were overall significant ($p < 0.0001$). Time needed to perform the TUG decreased considerably to 71.2% ± 18.0% over the course of the 8 weeks of therapy ($\bar{P} = 0.0004 \pm 0$). Significant differences in the time needed to perform the TUG were also found between baseline and 4-weeks assessment and 4-weeks and 8-weeks assessment ($\bar{P} = 0.0097 \pm 0.0132$ and $p = 0.0004$, respectively).

There were significant overall results in LEMS ($p < 0.0001$). Post hoc analysis showed significant ($\bar{P} = 0.0001 \pm 0$) increases in LEMS throughout the entire training phase, up to 120.5% ± 9.3% at the 8-weeks assessment. Post hoc analysis revealed a significant improvement in the strength of key muscles from baseline to 4 weeks ($\bar{P} = 0.0002 \pm 0.0001$) and 8 weeks ($\bar{P} = 0.0001 \pm 0$) of therapy and between 4 and 8 weeks of therapy ($p = 0.0054$).

The MAS of all study participants did not show any significant differences over the course of the therapy ($p = 0.2379$). However, in the 7 participants in whom spasticity was present (defined by a mean MAS > 4) at baseline (median at baseline: 16 (IQR 9.5 to 16)), a trend towards decreased spasticity (median: -3 (IQR-5.5 to -1.5)) was observed.

Two temporary conversions in the AIS (B to C and back to B and D to C and back to D) were detected during the therapy (onset, 4 weeks, 8 weeks) period [49]. The neurological level of injury remained within ± 2 segments referenced to baseline in all participants.

Follow-up examinations

Baseline and follow-up assessments (Fig. 3) were scheduled at the spinal cord injury center, while tests performed after 4 and 8 weeks of training were conducted at the participants' homes. Accordingly, the follow-up assessment was associated with significant travel efforts for the participants. Ten individuals attended the follow-up assessment. We performed a non-confirmatory descriptive subgroup analysis to search for differences in outcome measures between the groups of "follow-up attendees" and "non-attendees". Differences between these subgroups were found only in the WISCI II assessment with attendees of the follow-up visit showing 1) a much higher relative improvement at end of therapy (Fig. 5A) and 2) a much lower absolute baseline level than non-attendees (Fig. 5B).

Analysis of the WISCI II follow-up assessments shows that after the end of therapy, 1 subject further improved (9 to 16), 7 remained at the same level and 2 became worse (19 to 5, 16 to 11). Five months after therapy onset, 7 of the 10 follow-up visitors were less dependent on walking aids compared to baseline.

Patient reported outcome

Individuals were overall satisfied with their training experience (3.80 ± 0.85, Fig. 6A). The perception of safety during training was rated high, with a mean score of 4.20 ± 0.77. While movement patterns, foot stimulation and body position during training were rated as good, the simulation of physiological gait was rated as moderate.

Transfers on the device and back to the wheelchair, as well as fastening and releasing leg straps were rated as between moderate and easy (Fig. 6B), whereby transfer and fixation on the device were rated as to be slightly more difficult (3.58 ± 0.87) than releasing the straps and transfer back to the wheelchair (3.73 ± 1.0). Five individuals, among them two individuals with tetraplegia, declared the need for help in the process of mounting and/or dismounting the device.

Study participants were overall satisfied with the outcome of the therapy (Fig. 6C), indicated by a mean satisfaction score for the category "Therapy outcome" of 3.89 ± 1.49.

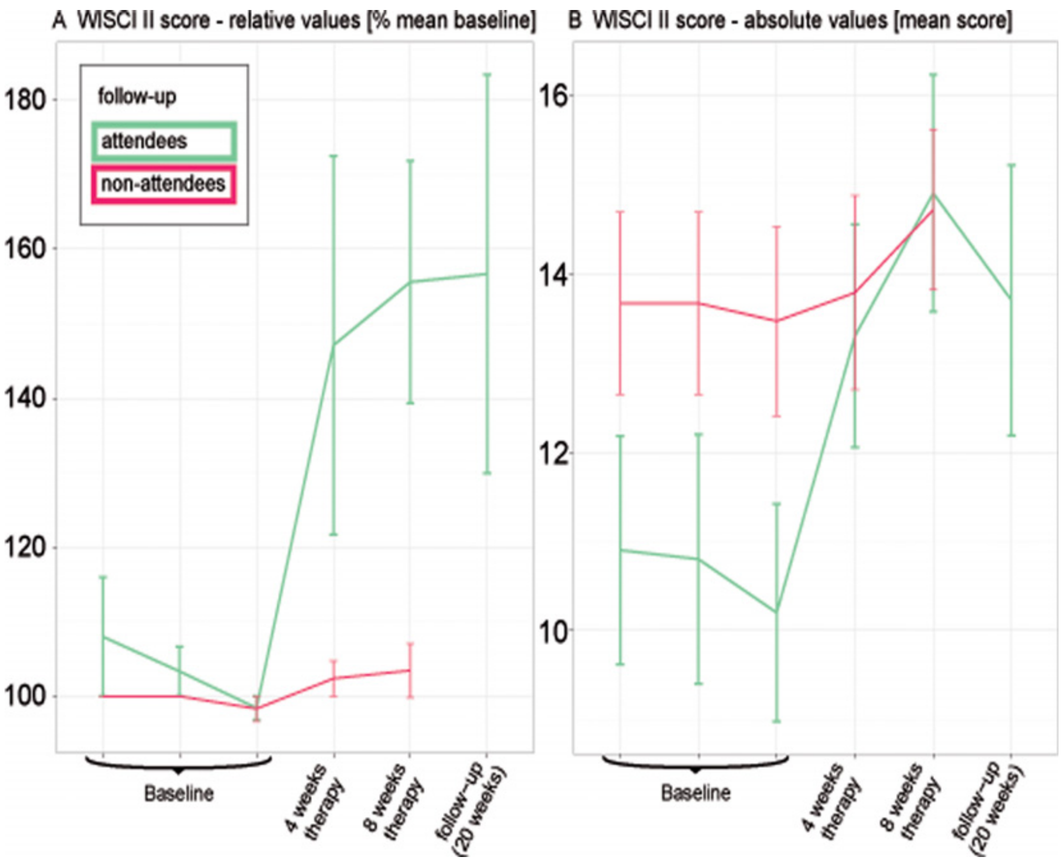

Fig 5. Analysis of the Walking Index for Spinal Cord Injury II (WISCI II) scores of the attendees of the follow-up visits. WISCI II scores displayed as (A) relative values ± standard error (SE) and (B) absolute values ± SE for participants (N = 10) who attended the follow-up assessment (green) and for participants (N = 15) who did not attend the follow-up assessment (red).

doi:10.1371/journal.pone.0119167.g005

Discussion

We investigated the safety and efficacy of 5 prototypes of the novel MoreGait robotic locomotor training device, which has been explicitly developed and designed for autonomous use in the home environment. This is to our knowledge the world's first locomotor training device dedicated to this purpose [50].

We found that home-based training with this compact device is feasible and effective, and could be handled well by the users. A study assessing the safety of the supervised application of the driven gait orthosis Lokomat in children and adolescents reported 5 adverse events requiring discontinuation of therapy in about 1.400 training sessions [51]. In more than 800 unsupervised training sessions of comparable duration with the MoreGait only one device-related adverse event occurred. In conclusion, the low incidence of device-related adverse events, the non-occurrence of any serious adverse event and the overall positive patient reports on safety and usability show that the MoreGait can be used safely and mainly independently in the home environment by sensorimotor impaired end users without continuous supervision by clinical experts.

The participants in our study with chronic iSCI showed no signs of neurological or functional recovery during the 4-week baseline period. This clearly demonstrates that the documented improvements following MoreGait training were therapy related and not spontaneous. The mean gain of 2.08 ± 3.82 levels in WISCI II clearly exceeds the recently reported clinically meaningful threshold of 1 level in iSCI [52].

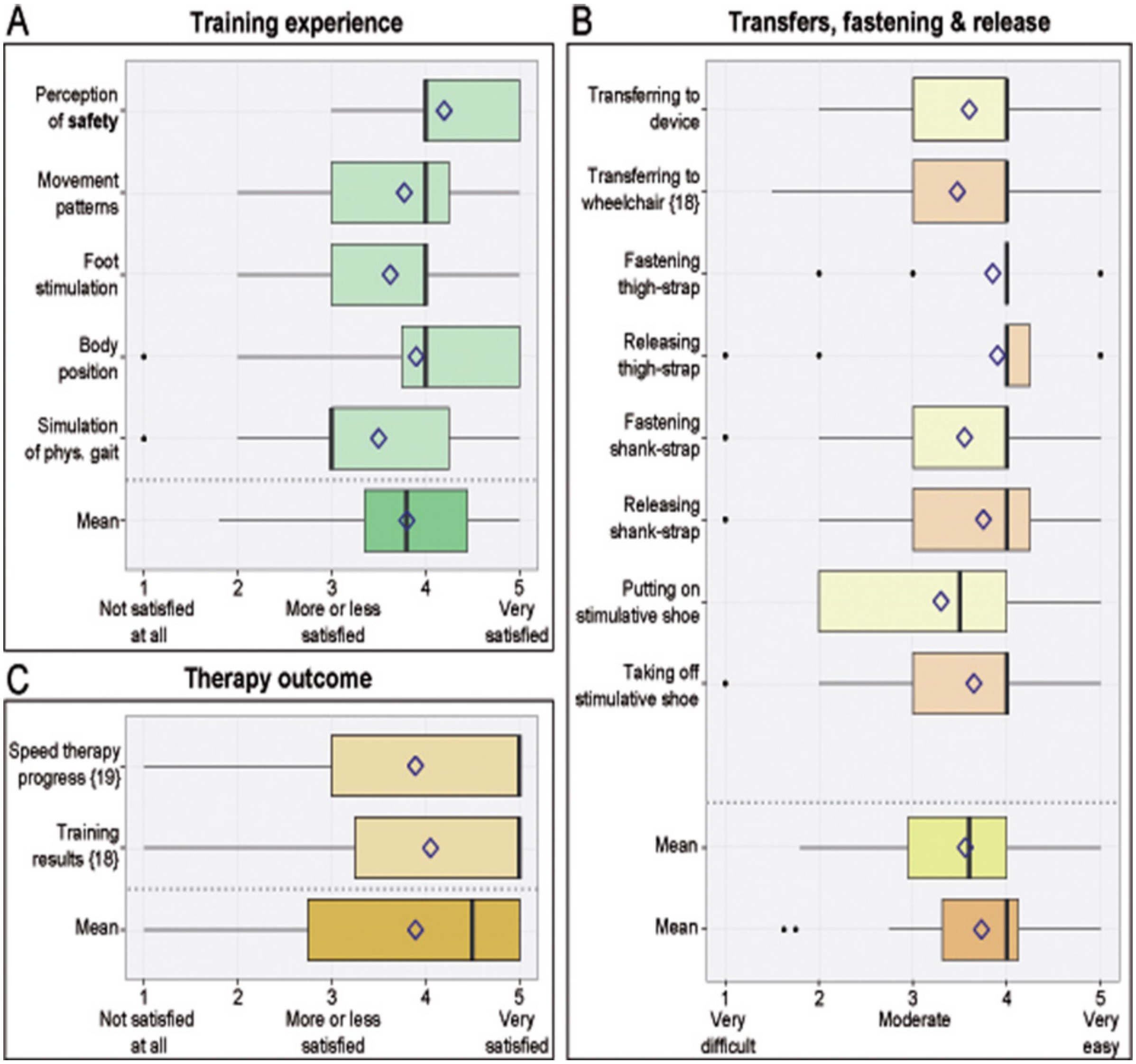

Fig 6. User survey results. Boxplots showing survey results on a 5 point scale for the categories (A) Training experience, (B) Transfers, fastening and release, and (C) Therapy outcome. The survey was completed by twenty of the twenty-five participants who finished the MoreGait study. Sample sizes are displayed in "{}", where they deviate from N = 20.

doi:10.1371/journal.pone.0119167.g006

The users benefited from the home-based robotic locomotion therapy in a variety of ways. The dependency on walking aids—a highly relevant issue for individuals with iSCI—was remarkably reduced in 9 participants after the MoreGait therapy. The importance of this aspect for the study participants is underlined by the outcome of the descriptive subgroup analysis of follow-up attendees vs. non-attendees. Attendees of the follow-up assessment had a much higher increase in WISCI II levels than non-attendees. The therapy-induced gain in WISCI II levels and *not* the absolute level at the end of therapy appears to influence participants' motivation level and, as a consequence, their willingness to participate in the last study assessment. The fact that the MoreGait therapy was home-based might have a negative impact on the general willingness to follow-up attendance compared to studies with inpatient interventions. However, to confirm the results from this descriptive subgroup analysis, a higher number of study participants needs to be included.

During the therapy period, the participants' walking ability increased considerably. This was reflected by improvements in short-distance gait speed (10MWT) by approximately 40%, endurance (6-MIN-TEST) by roughly 65% and standing up, turning, and sitting down (TUG) by around 30%. Those improvements were also seen in the 9 individuals, who needed less support by walking aids over the course of the therapy.

A direct comparison of the study results with other studies is very difficult due to differences in patient populations (type/severity of lesion, functional status, exclusion of spontaneous recovery,) and therapy regimens (frequency, duration) [53, 54]. However, to allow for direct comparison of results, the inclusion criteria and protocol selected for the MoreGait study were similar to those used for a study with the Lokomat [26]. The extent of improvement in walking speed and endurance achieved by MoreGait is comparable to that of supervised clinical gait rehabilitation with the Lokomat (10MWT: 56%, 6-MIN-TEST: 53%, TUG: 32%). However, a higher percentage of study participants was less dependent on walking aids after MoreGait training (MoreGait: 9/25, Lokomat: 2/21). Interestingly, participants in the MoreGait study increased gait speed and endurance during the therapy period in nearly linear fashion, indicating that MoreGait training performed for longer than 8 weeks might further improve locomotor function. Harkema et al. assessed the effects of intensive locomotor training, including step training using body-weight support and manual facilitation on a treadmill followed by overground assessment and community integration, in 196 individuals with chronic iSCI (approximately the same percentage of one third AIS C and two thirds AIS D participants as in our study) who underwent at least 20 locomotor training treatment sessions in outpatient rehabilitation centers [55]. Although the number of therapy sessions varied extensively, the group of chronic patients assessed within 1 to 3 years after trauma improved on average 0.11 ± 0.23 m/s in the 10MWT and 44 ± 71 m in the 6-MIN-TEST. Our participants improved to a similar extent (10MWT: 0.1 m/s; 6-MIN-TEST: 47 m), although they were not explicitly supervised by experienced therapists.

A number of studies utilizing body weight-supported training for improving walking in individuals with SCI have reported improvements in lower-limb strength in patients with chronic SCI that are in the range of our results [56, 57]. The improvements in walking ability could potentially be attributed only to the significant increase in LEMS. However, the motor scores of the proximal muscle groups, which are highly relevant for walking function, did not increase noticeably in the second half of the training period. Yet, locomotor function continued to improve to a similar extent compared to the first 4 weeks of training (Table 1). This points to an improvement in coordination rather than in muscle strength. Interestingly, the improvements in distal muscle strength, which mainly contributed to the increase in the total LEMS, are in the same range within the first and second half of the therapy period, indicating the high therapeutic relevance of the stimulative shoe.

There was a trend towards decreased spasticity at the end of therapy in study participants with a mean MAS at baseline greater than 4. However, this finding has to be interpreted very carefully due to the low reliability of the MAS to detect subtle changes in spasticity [58].

The following limitations of the study have to be considered: Besides screening of the internal medical database, study participants were recruited by advertising the study on the university hospital's website and in a magazine for people with disabilities focusing on individuals with SCI [34]. The recruitment procedure, together with the lack of reimbursement of travel expenses, may have contributed to a selection bias towards exceptionally motivated individuals. Considering the high dropout rate, the criterion for dropping out—less than 4x 30 minutes therapy time per week—was most likely too ambitious.

We did not document the type and focus of concomitant therapies and medication. Although study participants were asked not to modify their physical therapy, unsupervised

training program, or antispastic medication, it cannot be excluded that changes in the regimes of these therapies throughout the MoreGait training period contributed to the improvements. The MoreGait therapy was applied at home as an add-on therapy. Therefore, we cannot exclude that the gait improvements were simply caused by the higher training intensity. On the other hand, this regimen of use best reflects the intended application scenario, in which MoreGait is the key component for allowing a higher intensity of gait training at home. Randomized controlled studies are necessary in the future to show the superiority of the MoreGait training in comparison to other, more simple home-based therapies.

Due to the safety-driven design of the MoreGait the user is put in a semi-reclined position. The influence of this non-physiological posture during the locomotion therapy with MoreGait on balance needs to be determined in future studies.

While baseline and follow-up assessments were performed at the Spinal Cord Injury Center, the majority of the 4-weeks and 8-weeks assessments took part in the participant's home environment. While no influence of a community environment is reported on the 10MWT, positive effects are described on the gait endurance assessed by the 6-MIN-TEST [59]. This may lead to a systematic bias in the results of the 6-MIN-TEST obtained during the therapy period.

The findings of the present study demonstrate that a robotic device reduced to a technical minimum can be introduced into a feasible, safe and effective gait rehabilitation therapy at home and thus might influence future robotic gait-rehabilitation strategies. A randomized-controlled trial investigating the effects of MoreGait therapy in acute iSCI is currently underway. Other neurological disease conditions affecting locomotor function may also benefit from this kind of robotic therapy, and thus warrant future investigation. The MoreGait device represent a valuable platform for future investigations on systematic identification and ranking of the therapeutic impact of machine parameters like degree of foot loading, inclination of the backrest or the prolonged therapy time.

Conclusions

Robotic home-based locomotion therapy with MoreGait allows patients to continue high-frequency training of locomotor function based on principles of activation of spinal locomotor networks and of motor learning after discharge from rehabilitation centers. The functional improvements following 8 weeks of MoreGait therapy in individuals with chronic sensorimotor iSCI are well within the range of those achieved with complex locomotion robots used at hospitals [26]. The stimulative shoe provides the opportunity to investigate alternative foot-loading patterns (eg, gait phase-related vibrational patterns), which might be even more effective in activating the spinal locomotion network [28]. Of course, other neurological diseases affecting locomotor function may also benefit from this kind of robotic therapy, and thus warrant future investigation.

Supporting Information

S1 TREND Checklist. TREND statement checklist.
(PDF)

S1 Protocol. Trial Protocol.
(PDF)

S1 Table. P-values and confidence intervals. Overall p-values, as well as p-values and confidence intervals (ci) of the post-hoc comparisons of all baseline (BL) measurements, each BL with 4 weeks, and each BL with 8 weeks outcomes of the Walking Index for Spinal Cord Injury II (WISCI II), 10-Meter Walk Test (10MWT)—self-selected speed (sss) and maximum speed

(ms), six-minute walk test (6-MIN-TEST), Timed Up and Go Test (TUG) and lower extremity motor scores (LEMS) are listed chronologically. Also the mean and standard deviations of p-values of the comparison among all BL, BL with 4 weeks, and BL with 8 weeks are provided. All significant differences ($p < 0.05$) are marked in red.
(XLSX)

S2 Table. Data on patient demographics and of all study assessments. The top row contains the descriptor of each of the assessments. The rest of the rows of the table contain the assessment data of each study participant.
(XLSX)

S3 Table. Data of end user survey. The top row contains the items of the end user survey. Each of the rows of the rest of the table contains the answers of each end user to each of the items.
(XLSX)

Acknowledgments

The authors thank all study participants, as well as F. Degenhard, M. Niess, O. Betz, J. Nückles and W. Roth for their support in maintenance and repair of the MoreGait devices during the study.

Author Contributions

Conceived and designed the experiments: RR HP CS HJG MK EPH. Performed the experiments: HP MK. Analyzed the data: DS RR CS NW HJG. Contributed reagents/materials/analysis tools: MK EPH RR DS CS. Wrote the paper: RR DS HP CS HJG NW EPH MK.

References

1. Harkema SJ, Hillyer J, Schmidt-Read M, Ardolino E, Sisto SA, Behrman AL. Locomotor training: as a treatment of spinal cord injury and in the progression of neurologic rehabilitation. Arch Phys Med Rehabil. 2012; 93(9):1588–97. doi: 10.1016/j.apmr.2012.04.032 PMID: 22920456
2. Dietz V, Colombo G, Jensen L, Baumgartner L. Locomotor capacity of spinal cord in paraplegic patients. Ann Neurol. 1995; 37(5):574–82. PMID: 7755351
3. Schmidt RA, Lee TD. Motor Control and Learning; A Behavioral Emphasis. 4th Edition ed. Champaign, Illinois, USA Human Kinetics; 2005.
4. Wolpaw JR. The education and re-education of the spinal cord. Prog Brain Res. 2006; 157:261–80. PMID: 17167916
5. Grillner S, Zangger P. How detailed is the central pattern generation for locomotion? Brain Res. 1975; 88(2):367–71. PMID: 1148835
6. Pearson KG. Neural adaptation in the generation of rhythmic behavior. Annu Rev Physiol. 2000; 62:723–53. PMID: 10845109
7. Dimitrijevic MR, Gerasimenko Y, Pinter MM. Evidence for a spinal central pattern generator in humans. Ann N Y Acad Sci. 1998; 860:360–76. PMID: 9928325
8. Harkema S, Gerasimenko Y, Hodes J, Burdick J, Angeli C, Chen Y, et al. Effect of epidural stimulation of the lumbosacral spinal cord on voluntary movement, standing, and assisted stepping after motor complete paraplegia: a case study. Lancet. 2011; 377(9781):1938–47. doi: 10.1016/S0140-6736(11)60547-3 PMID: 21601270
9. Musselman KE, Yang JF. Loading the limb during rhythmic leg movements lengthens the duration of both flexion and extension in human infants. Journal of neurophysiology. 2007; 97(2):1247–57. PMID: 17151226
10. Musselman KE, Yang JF. Interlimb coordination in rhythmic leg movements: spontaneous and training-induced manifestations in human infants. Journal of neurophysiology. 2008; 100(4):2225–34. doi: 10.1152/jn.90532.2008 PMID: 18650307
11. Edgerton VR, Courtine G, Gerasimenko YP, Lavrov I, Ichiyama RM, Fong AJ, et al. Training locomotor networks. Brain Res Rev. 2008; 57(1):241–54. PMID: 18022244

12. Rossignol S, Frigon A. Recovery of locomotion after spinal cord injury: some facts and mechanisms. Annu Rev Neurosci. 2011; 34:413–40. doi: 10.1146/annurev-neuro-061010-113746 PMID: 21469957

13. Singh A, Balasubramanian S, Murray M, Lemay M, Houle J. Role of spared pathways in locomotor recovery after body-weight-supported treadmill training in contused rats. J Neurotrauma. 2011; 28 (12):2405–16. doi: 10.1089/neu.2010.1660 PMID: 21568686

14. Hultborn H, Nielsen JB. Spinal control of locomotion—from cat to man. Acta Physiol (Oxf). 2007; 189 (2):111–21. PMID: 17250563

15. Krakauer JW. Motor learning: its relevance to stroke recovery and neurorehabilitation. Curr Opin Neurol. 2006; 19(1):84–90. PMID: 16415682

16. Yang JF, Musselman KE. Training to achieve over ground walking after spinal cord injury: A review of who, what, when, and how. J Spinal Cord Med. 2012; 35(5):293–304. doi: 10.1179/2045772312Y.0000000036 PMID: 23031166

17. Dietz V. Locomotor training in paraplegic patients. Ann Neurol. 1995; 38(6):965. PMID: 8526474

18. Dobkin BH, Harkema S, Requejo P, Edgerton VR. Modulation of locomotor-like EMG activity in subjects with complete and incomplete spinal cord injury. J Neurol Rehabil. 1995; 9(4):183–90. PMID: 11539274

19. Wernig A, Muller S. Laufband locomotion with body weight support improved walking in persons with severe spinal cord injuries. Paraplegia. 1992; 30(4):229–38. PMID: 1625890

20. Colombo G, Wirz M, Dietz V. Driven gait orthosis for improvement of locomotor training in paraplegic patients. Spinal Cord. 2001; 39(5):252–5. PMID: 11438840

21. Hesse S, Werner C, Uhlenbrock D, von Frankenberg S, Bardeleben A, Brandl-Hesse B. An electromechanical gait trainer for restoration of gait in hemiparetic stroke patients: preliminary results. Neurorehabil Neural Repair. 2001; 15(1):39–50. PMID: 11527278

22. Dobkin BH, Duncan PW. Should body weight-supported treadmill training and robotic-assistive steppers for locomotor training trot back to the starting gate? Neurorehabil Neural Repair. 2012; 26(4):308–17. doi: 10.1177/1545968312439687 PMID: 22412172

23. Dobkin B, Apple D, Barbeau H, Basso M, Behrman A, Deforge D, et al. Weight-supported treadmill vs over-ground training for walking after acute incomplete SCI. Neurology. 2006; 66(4):484–93. PMID: 16505299

24. NSCISC. The 2006 Annual Statistical Report for the Model Spinal Cord Injury Care System: National SCI Statistical Center; 2006. Available from: www.uab.edu/NSCISC. Accessed 31 January 2015.

25. Hicks AL, Adams MM, Martin Ginis K, Giangregorio L, Latimer A, Phillips SM, et al. Long-term body-weight-supported treadmill training and subsequent follow-up in persons with chronic SCI: effects on functional walking ability and measures of subjective well-being. Spinal Cord. 2005; 43(5):291–8. PMID: 15685260

26. Wirz M, Zemon DH, Rupp R, Scheel A, Colombo G, Dietz V, et al. Effectiveness of automated locomotor training in patients with chronic incomplete spinal cord injury: a multicenter trial. Arch Phys Med Rehabil. 2005; 86(4):672–80. PMID: 15827916

27. Rupp R, Plewa H, Hofer EP, Knestel M. MotionTherapy@Home—a robotic device for automated locomotion therapy at home,. IEEE 11th International Conference on Rehabilitation Robotics—ICORR; Kyoto, Japan2009. p. 395–400.

28. Dietz V, Muller R, Colombo G. Locomotor activity in spinal man: significance of afferent input from joint and load receptors. Brain. 2002; 125(Pt 12):2626–34.

29. Layne CS, Forth KE, Baxter MF, Houser JJ. Voluntary neuromuscular activation is enhanced when paired with a mechanical stimulus to human plantar soles. Neurosci Lett. 2002; 334(2):75–8. PMID: 12435475

30. Sigrist R, Rauter G, Riener R, Wolf P. Augmented visual, auditory, haptic, and multimodal feedback in motor learning: a review. Psychon Bull Rev. 2013; 20(1):21–53. doi: 10.3758/s13423-012-0333-8 PMID: 23132605

31. Rupp R, Plewa H, Schuld C, Gerner HJ, Hofer EP, Knestel M. MotionTherapy@Home—First results of a clinical study with a novel robotic device for automated locomotion therapy at home. Biomed Tech (Berl). 2011; 56(1):11–21. doi: 10.1515/BMT.2010.051 PMID: 21080894

32. Kirshblum SC, Burns SP, Biering-Sorensen F, Donovan W, Graves DE, Jha A, et al. International standards for neurological classification of spinal cord injury (revised 2011). J Spinal Cord Med. 2011; 34 (6):535–46. doi: 10.1179/204577211X13207446293695 PMID: 22330108

33. Dittuno PL, Ditunno JF Jr. Walking index for spinal cord injury (WISCI II): scale revision. Spinal Cord. 2001; 39(12):654–6. PMID: 11781863

34. Rupp R. Motorisches Training für inkomplett Querschnittgelähmte. Paraplegiker. 2009; 27(1):52–4.

35. Knestel M, Hofer EP, Klee Barillas S, Rupp R. The Artificial Muscle as an Innovative Actuator in Rehabilitation Robotics. 17th World Congress of the International Federation of Automatic Control; Seoul, Korea. 2008. p. 773–8.
36. Knestel M, Hofer EP, Rupp R. Model-Based Feedback Control of a Rehabilitation Robot. 2nd International Convention on Rehabilitation Engineering & Assistive Technology; Bankok, Thailand. 2008. p. 175–8.
37. Graham JE, Karmarkar AM, Ottenbacher KJ. Small sample research designs for evidence-based rehabilitation: issues and methods. Arch Phys Med Rehabil. 2012; 93(8 Suppl):S111–6.
38. Marino RJ, Scivoletto G, Patrick M, Tamburella F, Read MS, Burns AS, et al. Walking index for spinal cord injury version 2 (WISCI-II) with repeatability of the 10-m walk time: Inter- and intrarater reliabilities. American journal of physical medicine & rehabilitation / Association of Academic Physiatrists. 2010; 89 (1):7–15.
39. van Hedel HJ, Wirz M, Dietz V. Assessing walking ability in subjects with spinal cord injury: validity and reliability of 3 walking tests. Arch Phys Med Rehabil. 2005; 86(2):190–6. PMID: 15706542
40. van Hedel HJ, Wirz M, Curt A. Improving walking assessment in subjects with an incomplete spinal cord injury: responsiveness. Spinal Cord. 2006; 44(6):352–6. PMID: 16304565
41. Kirshblum SC, Waring W, Biering-Sorensen F, Burns SP, Johansen M, Schmidt-Read M, et al. Reference for the 2011 revision of the International Standards for Neurological Classification of Spinal Cord Injury. J Spinal Cord Med. 2011; 34(6):547–54. doi: 10.1179/107902611X13186000420242 PMID: 22330109
42. Schuld C, Wiese J, Franz S, Putz C, Stierle I, Smoor I, et al. Effect of formal training in scaling, scoring and classification of the International Standards for Neurological Classification of Spinal Cord Injury. Spinal Cord. 2013; 51(4):282–8. doi: 10.1038/sc.2012.149 PMID: 23184026
43. Schuld C, Wiese J, Hug A, Putz C, Hedel HJ, Spiess MR, et al. Computer implementation of the international standards for neurological classification of spinal cord injury for consistent and efficient derivation of its subscores including handling of data from not testable segments. J Neurotrauma. 2012; 29 (3):453–61. doi: 10.1089/neu.2011.2085 PMID: 21933016
44. Smith AW, Jamshidi M, Lo SK. Clinical measurement of muscle tone using a velocity-corrected modified Ashworth scale. American journal of physical medicine & rehabilitation / Association of Academic Physiatrists. 2002; 81(3):202–6.
45. Demers L, Weiss-Lambrou R, Ska B. Item analysis of the Quebec User Evaluation of Satisfaction with Assistive Technology (QUEST). Assistive technology: the official journal of RESNA. 2000; 12(2):96–105. PMID: 11508406
46. R Core Team. R: A Language and Environment for Statistical Computing. Vienna, Austria: R Foundation for Statistical Computing; 2012.
47. Benjamini Y, Hochberg Y. Controlling the false discovery rate: a practical and powerful approach to multiple testing. Journal of the Royal Statistical Society Series B (Methodological). 1995:289–300.
48. Shea JD. Pressure sores: classification and management. Clin Orthop Relat Res. 1975;(112):89–100.
49. Spiess MR, Muller RM, Rupp R, Schuld C, Group E- SS, van Hedel HJ. Conversion in ASIA impairment scale during the first year after traumatic spinal cord injury. J Neurotrauma. 2009; 26(11):2027–36. doi: 10.1089/neu.2008-0760 PMID: 19456213
50. Hofer EP, Knestel M, Rupp R, Vosseler M.Device for moving limbs. European Patent No. EP000001959909A1. 2008.
51. Borggraefe I, Klaiber M, Schuler T, Warken B, Schroeder SA, Heinen F, et al. Safety of robotic-assisted treadmill therapy in children and adolescents with gait impairment: a bi-centre survey. Developmental neurorehabilitation. 2010; 13(2):114–9. doi: 10.3109/17518420903321767 PMID: 20222772
52. Burns AS, Delparte JJ, Patrick M, Marino RJ, Ditunno JF. The reproducibility and convergent validity of the walking index for spinal cord injury (WISCI) in chronic spinal cord injury. Neurorehabil Neural Repair. 2011; 25(2):149–57. doi: 10.1177/1545968310376756 PMID: 21239706
53. Morawietz C, Moffat F. Effects of Locomotor Training After Incomplete Spinal Cord Injury: A Systematic Review. Arch Phys Med Rehabil. 2013.
54. Mehrholz J, Kugler J, Pohl M. Locomotor training for walking after spinal cord injury. Cochrane Database Syst Rev. 2012; 11:CD006676. doi: 10.1002/14651858.CD006676.pub3 PMID: 23152239
55. Harkema SJ, Schmidt-Read M, Lorenz DJ, Edgerton VR, Behrman AL. Balance and ambulation improvements in individuals with chronic incomplete spinal cord injury using locomotor training-based rehabilitation. Arch Phys Med Rehabil. 2012; 93(9):1508–17. doi: 10.1016/j.apmr.2011.01.024 PMID: 21777905
56. Behrman AL, Harkema SJ. Locomotor training after human spinal cord injury: a series of case studies. Phys Ther. 2000; 80(7):688–700. PMID: 10869131

57. Field-Fote EC. Combined use of body weight support, functional electric stimulation, and treadmill training to improve walking ability in individuals with chronic incomplete spinal cord injury. Arch Phys Med Rehabil. 2001; 82(6):818–24. PMID: 11387589

58. Craven BC, Morris AR. Modified Ashworth scale reliability for measurement of lower extremity spasticity among patients with SCI. Spinal Cord. 2010; 48(3):207–13. doi: 10.1038/sc.2009.107 PMID: 19786977

59. Olmos LE, Freixes O, Gatti MA, Cozzo DA, Fernandez SA, Vila CJ, et al. Comparison of gait performance on different environmental settings for patients with chronic spinal cord injury. Spinal Cord. 2008; 46(5):331–4. PMID: 17923845
